# Common and distinct organ and stress responsive transcriptomic patterns in *Oryza sativa *and *Arabidopsis thaliana*

**DOI:** 10.1186/1471-2229-10-262

**Published:** 2010-11-24

**Authors:** Reena Narsai, Ian Castleden, James Whelan

**Affiliations:** 1 ARC Centre of Excellence in Plant Energy Biology, MCS Building M316 University of Western Australia; 2 Centre for Computational Systems Biology, 35 Stirling Highway, Crawley 6009, Western Australia, Australia

## Abstract

**Background:**

*Arabidopsis thaliana *is clearly established as the model plant species. Given the ever-growing demand for food, there is a need to translate the knowledge learned in Arabidopsis to agronomically important species, such as rice (*Oryza sativa*). To gain a comparative insight into the similarities and differences into how organs are built and how plants respond to stress, the transcriptomes of Arabidopsis and rice were compared at the level of gene orthology and functional categorisation.

**Results:**

Organ specific transcripts in rice and Arabidopsis display less overlap in terms of gene orthology compared to the orthology observed between both genomes. Although greater overlap in terms of functional classification was observed between root specific transcripts in rice and Arabidopsis, this did not extend to flower, leaf or seed specific transcripts. In contrast, the overall abiotic stress response transcriptome displayed a significantly greater overlap in terms of gene orthology compared to the orthology observed between both genomes. However, ~50% or less of these orthologues responded in a similar manner in both species. In fact, under cold and heat treatments as many or more orthologous genes responded in an opposite manner or were unchanged in one species compared to the other. Examples of transcripts that responded oppositely include several genes encoding proteins involved in stress and redox responses and non-symbiotic hemoglobins that play central roles in stress signalling pathways. The differences observed in the abiotic transcriptomes were mirrored in the presence of *cis*-acting regulatory elements in the promoter regions of stress responsive genes and the transcription factors that potentially bind these regulatory elements. Thus, both the abiotic transcriptome and its regulation differ between rice and Arabidopsis.

**Conclusions:**

These results reveal significant divergence between Arabidopsis and rice, in terms of the abiotic stress response and its regulation. Both plants are shown to employ unique combinations of genes to achieve growth and stress responses. Comparison of these networks provides a more rational approach to translational studies that is based on the response observed in these two diverse plant models.

## Background

*Arabidopsis thaliana *is clearly established as the pre-eminent model plant species [1,2]. A major challenge in plant biology research is to translate the knowledge learned in Arabidopsis to agronomically important species in an attempt to address the growing demand for food to feed the 9 billion people by 2050, requiring an increase of 70% in food production [3,4]. The application of various genomic approaches directly to crop species, such as observed with the sequencing of the maize genome [5], and the power of the new generation of sequencing technologies means that genomic wide studies on crop species are now feasible [6]. The use of marker assisted selection (MAS) and mapped based cloning (MBC) have also been successfully applied in a variety of cereal plants to produce field varieties with desired traits [4,7,8]. However, despite the application of these technologies and methodologies, it does not nearly match the knowledge directly obtained from model species. Additionally, the high co-linearity that exists between cereal genomes allows comparative genomic approaches to be used, so that genes identified in one cereal or wild-type relatives can be quickly identified and selected in crop and/or other cereal species [8]. However, it is important to note that these approaches are component based and thus fail to take into account how different components can interact in a system.

It is obvious to state that the genome is the same in all cells in an individual but yet can produce different 'phenotypes' due to differentiation and development. The combinatorial regulation of gene expression has the potential to produce almost endless combinations of novel solutions to challenges in the environment. There are many examples in evolution where the same phenotype can be achieved via different mechanisms [9], such as bract suppression in monocots and dicots [10]. Similar outcomes or traits can be achieved using different approaches, a good example of this is the evolution of C4 photosynthesis in plants, that has arisen independently many times in angiosperm plants [11]. Transcriptional networks change rapidly with respect to evolutionary time, binding sites for transcription factors can be changed in a small number of mutations that can alter a transcriptional network [12]. Organisms closely related in evolutionary terms can have different motifs to regulate similar sets of genes due to the fact that different environments select different combinatorial networks that are required to regulate genes in each environment [12]. Comparative systems biology scale approaches reveal that changes in phenotype are often a result of small changes in the way components interact or that similar biological outcomes can be achieved by networks of different components [13].

Transcriptional networks are the dynamic expression of the gene complement of an organism. As polyploidisation is a major speciation mechanism in plants this has the potential to cause large-scale changes in the gene complement. In particular it seems that genes encoding regulatory factors, e.g. transcription factors, are preferentially retained after duplication events and the divergence of these regulatory factors plays a role in diversification in flowering plants [14]. This diversification of regulatory factors has the potential to results in large scale re-arrangements in transcriptome signatures, and many studies carried out at the level of specific gene families reveal species specific functions. An analysis of both lectin and lignin biosynthesis genes between Arabidopsis and rice concluded that lineage-specific expansion has occurred that would result in species-specific traits [15,16]. Likewise, at the level of signalling, genes involved in controlling flowering time [17] and genes encoding F-Box protein involved in substrate-recognition for ubiquitin ligases [18] have diverged. Similarly, at the transcriptional level, analysis of transcription factor families, such as the NAC and bHLH families across various plant species has revealed species specific activities of these, ranging from developmental to stress responses [19,20]. Given that genome duplication, chromosomal duplication and differential loss of duplicated genes has resulted in a relatively rapid evolution of plant genomes, ~140 million years of divergence between monocots and eudicots, compared to hundreds of millions of years of conservation between vertebrate genomes, this has resulted in different sets of genes that form the base of transcriptional networks in plants [11].

In order to gain an overview of how transcriptional networks vary between plants, an analysis of transcriptome profiles for similar data sets in rice and Arabidopsis was carried out with the view to investigate the level of similarly in transcriptional networks across organs and in response to abiotic stress. This would provide a genome wide overview, rather than a family-by-family comparison, of how plants grow and respond to the environment. Although Arabidopsis and rice represent a wide phylogenetic gap of ~140 million years in plant evolution [21], common responses are likely to be conserved across many plant species, while distinct responses are a signpost to where divergence has occurred [22]. Both provide insight into how organs are formed and grow, and how plants respond to the environment. These analyses will provide a useful resource for examining how similar outcomes can be achieved using different combinations of genes. These findings will provide a more rational approach to choosing candidates for translational research between species, where successes in achieving outcomes are often reported [23], yet the failure rate is unknown.

## Results

### Global analysis of transcriptome datasets

Although a variety of array platforms are used for transcriptome analysis in rice [24-26], the broadest collection of datasets is available on the Affymetrix platform. This is advantageous as it is the same platform as used for the Arabidopsis AtGenExpress project, so all parameters for parallel array comparisons could be identical with respect to statistical analysis. In addition, the range of experiments in rice is now also large, with organ, development, abiotic (heat, cold, drought salt), biotic (fungal, bacterial, viral) and hormone treatment experimental designs available. Although Arabidopsis is the pre-eminent plant model, rice is a close second. Arabidopsis is a eudicot and thus is separated by ~ 140 million years from monocot plants [21]. The major cereal crops *Triticum, Hordeum, Oryza, Sorghum, and Zea *are all monocot plants that supply over half of the calorific needs of the world population [27]. Given this demand, these cereals are grown all over the world and are therefore subject to vastly differential environmental conditions. Thus, a comparison between Arabidopsis, the most widely studied plant model, and rice, the model cereal, can provide information that may be beneficial to the major food producing crop plants.

**Figure 1:**
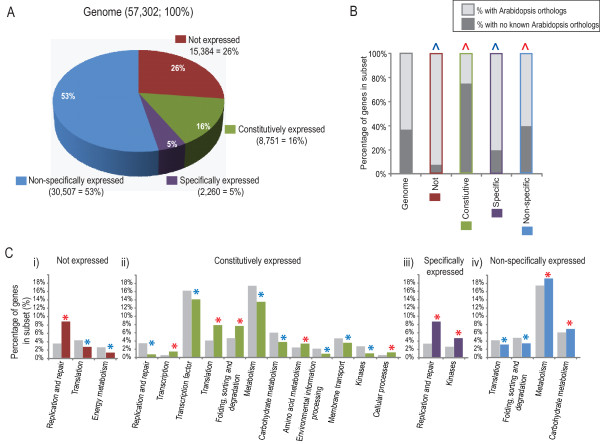
**Global expression analysis of rice transcriptome datasets**. **A) **Expression of the 57,302 probesets defined as the genome were classified as always expressed, never expressed, showing specific expression or non-specific expression based on MAS5.0 normalised microarray data. A probeset was considered to be expressed if it was called present in >/= 2 replicates in at least one sample. B) The proportion of rice transcripts that have Arabidopsis orthologues (determined by Inparanoid database v7.0) is indicated by the darker shade of colour for each subset. Significant (z-score, p < 0.01) enrichment/depletion of orthologues in each sub-set compared to the genome proportion is denoted with a red/blue ^, respectively. **B) **Analysis of FUNcational CATalogue (FUNCAT) was carried out on each set of genes. Subsets that were enriched or depleted in that FUNCAT compared to the genome are indicated by the red or blue asterisk respectively.

To carry out multi-array expression analysis for rice, normalised data from all 366 rice microarrays, representing 129 biological samples, were collated together and a probeset was considered to be expressed in a particular tissue/sample if at least 2 replicates for that sample showed statistically significant present calls (p < 0.05) [28]. The number of genes called present in this way followed the same distribution pattern as was expected with a large number of probesets seen at each tail of the distribution (Additional file 1, Figure S1) [29]. The microarray experiments included in these analyses are outlined in Table 1 (Additional file 2, Table S1). More than 41,000 unique probesets were expressed across one or more of the samples, with an average of ~24,000 probesets expressed per sample. Analysis of the 129-sample expression data matrix revealed differences in the global expression pattern across all the rice microarrays (Figure 1). In order to compare this to Arabidopsis, all rice probesets with TIGR identifiers were matched to Arabidopsis orthologues and the proportion of these with Arabidopsis orthologues are indicated by the darker shade of colour for each subset (Figure 1). It can be seen that only 37% of rice genes represented on the microarray had Arabidopsis orthologues (based on the Inparanoid Orthologue Database [30]). In contrast, reverse comparison revealed that 61% of all Arabidopsis genes had rice orthologues, indicating that a much larger proportion of non-orthologous genes exist in rice compared to Arabidopsis.

**Table 1:** Overview of the 366 Affymetrix rice genome microarrays and 177 Affymetrix Arabidopsis genome arrays used for the global analysis in this study.

Sample details	GEO/AT-EXP	Rep	No. Arrays	Tissue	Publications
**Development - Rice**					

Dry seed and aerobic germination (up to 24 h) cv. Amaroo	E-MEXP-1766	3	15	Dry and germinating seed	[83]
Dry seed and anaerobic germination (up to 24 h) and switch conditions cv. Amaroo	E-MEXP-2267	3	36	Imbibed seed	[84]
Aerobic and anaerobic grown coleoptiles cv. Nipponbare	GSE6908	2	4	Coleoptile	[85]
Embryo, endosperm, leaf and root from 7-d seedling, 10 d seedling cv. Zhonghua	GSE11966	2	10	Embryo, endosperm, leaf and root from 7-d seedling, 10-d seedling	[86]
Stigma, Ovary cv. Nipponbare	GSE7951	3	6	Stigma, ovary	[87]
Mature leaf, young leaf, semi apical meristem, inflorescence, seed cv. IR64	GSE6893	3	45	Mature leaf, young leaf, semi apical meristem, inflorescence, seed	[39]

**Abiotic stress - Rice**					

Drought, salt, cold stress cv. IR64	GSE6901	3	12	Seedling	[39]
Heat stress cv. Zhonghua	GSE14275	3	6	Seedling	[71]
Salt stress on 2 cultivars; indica, FL478 (salt tolerant), indica, IR29 (salt sensitive)	GSE3053	3	11	Crown and growing point	[88]
Salt stress on 4 cultivars; japonica, m103 (salt sensitive), indica, IR29 (salt sensitive), japonica, Agami (salt tolerant), indica, IR63731 (salt tolerant)	GSE4438	3	24	Panicle initiation stage	[89]
Salt stress on root using 4 cultivars; FL478 (salt tolerant), IR29 (salt sensitive), IR63731 (salt tolerant), Pokkali (salt tolerant)	GSE14403	3	23	Root	-
Fe and P treatments cv. Nipponbare	GSE17245	2	16	Root	[90]
Arsenate treatment cv. Azucena	GSE4471	3	12	Seedling	[91]
Physical stress at roots tips cv. Bala	GSE10857	3	12	Root tip	[92]

**Biotic stress - Rice**					

*S. Hermonthica *plant parasite infection cv. Nipponbare (resistant), IAC165 (susceptible)	GSE10373	2	24	Root	[93]
*M. grisea *blast fungus infection cv. Nipponbare	GSE7256	2	8	Leaf	[94]
Rice stripe virus infection cv. WuYun3, KT95-418	GSE11025	3	12	Seedling	-
Infection with bacteria *X. Oryzae *pv. oryzicola and oryzae cv. Nipponbare	GSE16793	4	60	Whole-plant tissue	-

**Hormone treatment - Rice**					

Cytokinin treatment on root and leaf cv. Nipponbare	GSE6719	3	24	Root, 2-week old seedlings	[95]
Indole-3-actetic acid and benzyl aminopurine treatment cv. IR64	GSE5167	2	6	Seedling	[96]

**Development - Arabidopsis**					

AtGenExpress developmental tissue microarray dataset (wildtype only)	E-AFMX-9	3	147	Wild type, different tissues	[97]

**Abiotic stress - Arabidopsis**					

AtGenExpress global stress expression dataset (wildtype)	GSE5620, GSE5624, GSE5623, GSE5621, GSE5628	2	30	Wild type, shoots	[80]

The microarray experiments are classified as development/tissue, abiotic stress, biotic stress or hormone treatment respectively depending on the purpose of the experiment. For each microarray set in each experiment an experimental description is given with the respective cultivar (cv.) indicated, public Gene Expression Omnibus (GSE) identifier or MIAME Genexpress identifier (E-MEXP), the number of biological replications carried out (Reps), the number of arrays carried out in that experiment, the tissues analysed are described and a reference (if published) is shown.

This analysis of rice global expression (based on microarrays) revealed that 26% of all probesets were never expressed, 16% were expressed in all samples (constitutively expressed), 5% were expressed exclusively in only 1 sample (showed specific expression) and 53% were expressed in between 2 to 128 samples (non-specifically expressed) (Figure 1A). In order to determine if there was a relationship between rice gene expression and gene orthology, it was determined how many genes in each subset had existing Arabidopsis orthologues and this was compared the genome percentage (37% of rice genes in the genome have Arabidopsis orthologues; Figure 1B). Notably, for the genes in each of these sub-sets, significant differences in the proportion of Arabidopsis orthologues were noted compared to the genome (Figure 1B). It could be seen that the genes which were never expressed and those showing specific expression had significantly lower proportions (z-score, p < 0.01) of Arabidopsis orthologues than the genome (8% and 20% respectively, compared to 37% in the genome; Figure 1B). In contrast, significantly higher proportions (z-score, p < 0.01) of genes that showed constitutive expression and those showing non-specific expression had orthologues in Arabidopsis (75% and 40%, respectively compared to the genome), implying a more conserved requirement for the expression of these genes in both species.

A Functional Catalogue (FUNCAT) analysis of the transcripts in each subset (Always, Never, Specific and Non-specific) was carried out based on predicted protein function (Figure 1C). The results reveal statistically significant (p < 0.01) differences in the representation of functional catalogues within each subset (Figure 1C, Additional file 2, Table S2). The subset of probesets that were never expressed were found to be significantly enriched in transcripts encoding replication and repair functions as well as environmental information processing/cellular processes (Figure 1C). It is important to note this subset is likely to include transcripts which are too low to be detected by microarray studies, thus this "never expressed" refers strictly to microarrays and it is likely that a large number of these transcripts are expressed and could possibly be detected by quantitative RT-PCR (qRT-PCR), which is known to be sensitive enough to detect low abundance transcripts such as transcription factors [31]. Nonetheless, it is interesting to note that this group was also significantly depleted in transcripts encoding proteins involved in core cellular functions such as translation functions (Figure 1C). This finding is complemented by finding in the "constitutively expressed" subset, which was significantly enriched (p < 0.01) in amino acid metabolism, transcription, translation, folding, sorting and degradation and cellular processes (Figure 1). Together, these findings suggest that transcripts encoding proteins necessary in any cell, such as those encoding transcription and translation functions, are consistently required, regardless of cell type or stress and thus appear to be expressed in all tissues at a high enough level to be detected by microarrays. This is also supported by the finding that more than double the proportion (76%) of genes that were constitutively expressed had orthologues in Arabidopsis (Figure 1B). Overall, these finding suggests that the fundamental processes of transcription, translation and protein folding would be expected to be well conserved between Arabidopsis and rice.

### Tissue/Development specific transcriptomic signatures in Arabidopsis and rice

**Figure 2:**
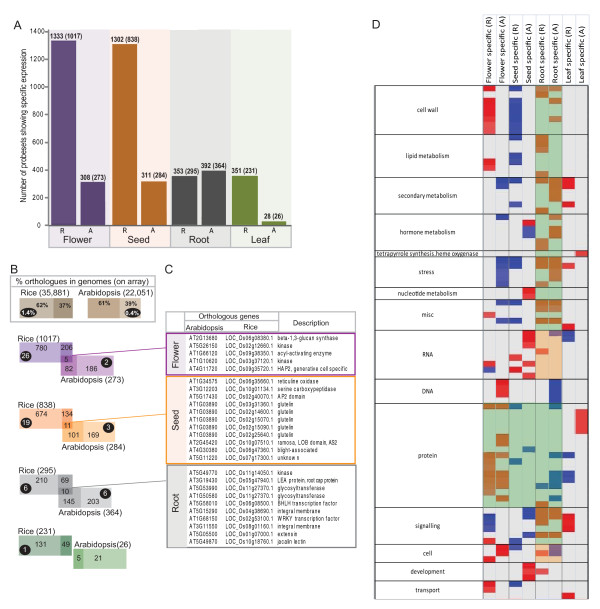
**Summary of transcripts that display development or tissue specific detection**. **A) **For each tissue and/or developmental stage (seed, leaf root and flower) the number of probesets expressed exclusively in one or more of the developmental stages for that tissue is shown e.g. present at one or more stage of inflorescence. The numbers in brackets indicates the number of probesets with known TIGR6 identifiers for rice or known TAIR9 AGIs for Arabidopsis. **B) **For each of the tissues, the probesets with annotated TIGR identifiers or AGIs for rice and Arabidopsis respectively, were analysed for orthologues that displayed the same type of restricted expression profile. Orthologues were determined based on the Inparanoid database v7.0 [30]. Overlap in the Venn diagram indicates the number of genes that were orthologous and tissue specific in both species e.g. 5 orthologous, flower specific genes. The number in the darker shades represent the number of orthologues that exist, but are not tissue specific in the respective other species. Lastly, the number in the lightest shade represents the number of genes that have no orthologues, with the number of lineage specific genes shown in the black circle (lineage specific was defined in [32]). **C) **The AGI, TIGR identifiers and description of each gene in the overlapping sets are also shown. **D) **PageMan analysis of the genes showing tissue specific expression. For each set of genes that showed tissue specific expression (e.g. all 231 leaf specific genes for rice), statistical analysis of over-represented functional categories was carried out using the Fisher method. Functional categories that did not show significant changes were collapsed for display. Statistical significances are represented by a false colour heat map (up, red; down, blue) where a z-score of 1.96 represents a p-value of 0.05.

Having established the basic transcriptome pattern of rice, a direct comparison to analogous datasets in Arabidopsis was possible. Firstly, the organ specific transcriptome for seed, leaf, root and flower was defined for rice and Arabidopsis by analysis of the microarrays were that were carried out using these organ types (Table 1; Additional file 2, Table S3). The same criteria were used to determine the specific nature of expression in both rice and Arabidopsis, where a probeset was considered to be organ specific if expressed in one or more conditions for that organ type, e.g. if a probeset was present in young leaf and/or mature leaf and in no other organs, it was defined as leaf specific. The number of root specific probesets expressed in Arabidopsis (392) was found to be comparable to rice (353) (Figure 2A). In contrast, the number of seed, leaf and flower specific genes Arabidopsis was less than a third of the number of respective organ specific genes in rice (Figure 2A). Given that the organ sets selected were broad in nature and that similar sets of genes were observed to be root specific in Arabidopsis and rice, this implies that the larger gene content of rice compared to Arabidopsis (50,000 (TIGR6) verse 35,000 (TIGR8) respectively) has undergone sub-functionalisation and thus, there are more genes that display specific expression patterns in rice.

To gain insight into this, all rice genes (with TIGR identifiers) and all Arabidopsis genes (with AGIs) were compared. Firstly, the number of genes showing organ specific expression was determined (e.g. 1017 flower specific genes in rice) and the number of existing orthologues within these sets were calculated and shown within the inner darker block within each set, e.g. 211 of the 1017 flower specific genes in rice had Arabidopsis orthologues, but only 6 were both orthologous and showing the flower specific response in (Figure 2B). Interestingly, it was seen that for all genes showing specific expression in each of the 4 tissues analysed in both species, there was a significantly smaller (p < 0.01) proportion of orthologues present in each of these sets compared to the proportions in the respective genomes (Figure 2B). In addition, of the 502 rice lineage specific genes [32] expressed on the rice microarray (making up 1.4% of the genome), a significantly larger proportion of these were present in the flower specific set (p < 0.01) and seed specific set (p < 0.02) in rice (26 genes; 2.5% and 19 genes; 2.2% respectively compared to 1.4% in the genome; Figure 2B). Similarly, a significant over-representation of Arabidopsis lineage specific genes was observed in the root specific set (6 genes; 1.6% vs. 0.4% in the genome; Figure 2B). Analysis of the orthologous genes that displayed tissue specific expression between both species indicated all five flower specific genes were involved in male gametophyte production, viability or pollen tube growth (Figure 2C). In the case of seed specific genes, genes encoding endomembrane proteins were dominant, whilst genes involved in cell wall growth were prominent in the root specific set (Figure 2C).

While the overlap between the organ specific genes was low in terms of gene orthology, it may be argued that direct orthology is too strict to reveal similar processes and that the organ specific genes in both species may be more similar if defined in terms of function. Thus, all tissue specific genes (regardless of orthology) were analysed based on the over/under-representation of functional categories using the PageMan software [33] (Figure 2D). In this comparison, greater similarities were seen for the over/under-representation of functional categories for some organs, such as roots, where many root specific transcripts were enriched in the same functional groups in both Arabidopsis and rice (green shade, Figure 2D). However, even in root, certain processes/functions showed differences in enrichment, such as for 'cell' and 'RNA' functions (orange shade, Figure 2D). In contrast, with the exception of 'protein' related functions, there was little overlap in the functional representation across Arabidopsis and rice for flower and seed specific gene sets (Figure 2D). Similarly, little functional overlap was seen between the Arabidopsis and rice leaf specific sets, with opposite over/under-representation even observed for genes encoding protein related functions (Figure 2D).

### Transcriptomic responses under abiotic stress for rice and Arabidopsis

**Figure 3:**
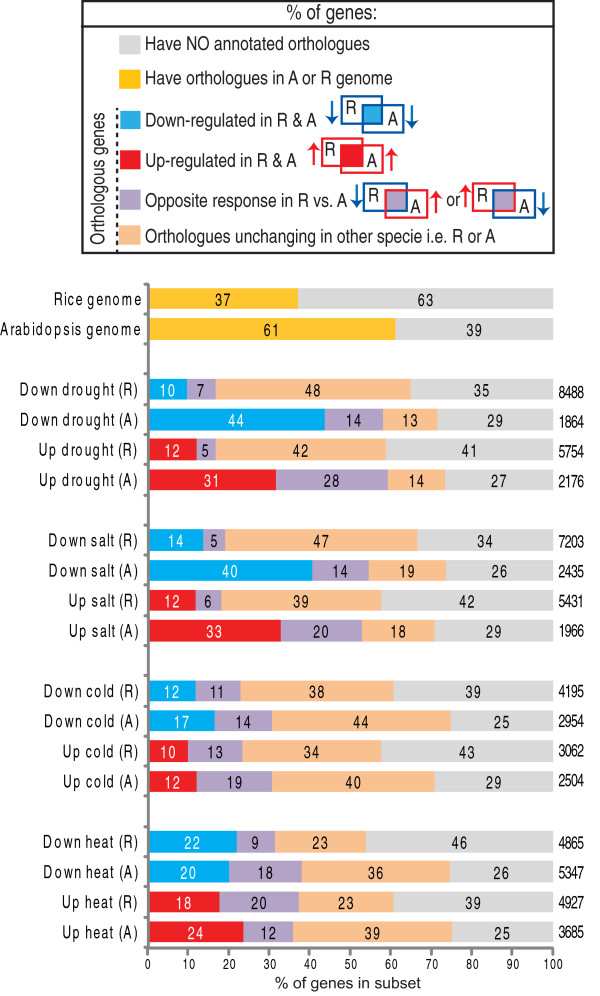
**Defining common and exclusive stress responsive genes**. The number of genes significantly (p < 0.05, PPDE > 0.96) differentially expressed in rice and Arabidopsis under drought, salt, cold and heat treated plants are shown on the right of each column. For each stress responsive sub-set of genes, the proportion of transcripts that i) have orthologous genes responding in a similar way (Up/Down-regulated in A & R; red/blue), ii) have orthologous genes responding in an opposite manner (e.g. up-regulated in rice and down-regulated in Arabidopsis; denoted Opposite response; purple), iii) have orthologous genes unchanging in the respective other species and iv) do not have orthologues (grey) is shown. The total number of genes in each sub-set is also shown next to each of the bars.

Analysis of the abiotic transcriptomes of Arabidopsis and rice in parallel enabled the identification of similarities and differences in the response to abiotic stress (Table 1). For the drought and salt responsive subsets, the number of differentially expressed genes (DEGs) in rice was much greater than in Arabidopsis, with over ~12,000 DEGs in rice compared to ~4,000 in Arabidopsis (Figure 3; Additional file 2, Table S4 and S5). In contrast, the number of DEGs following the cold and heat treatments was more comparable with 9,792 and 7, 257 genes differentially expressed in rice compared to 9, 032 and 5, 458 in Arabidopsis, respectively (Figure 3). These subsets of DEGs were then analysed to determine the proportion of genes that were known to have Arabidopsis/rice orthologues. It was observed that over 70% of Arabidopsis genes and over 55% of the rice genes in each differentially expressed subset have orthologues in rice and Arabidopsis, respectively, which is significantly (p < 0.01) larger than the proportion of orthologues in the genomes (61% and 37%, respectively) (Figure 2B and Figure 3). However, only 10%-14% of all rice DEGs in the drought and salt responsive subsets had orthologues in Arabidopsis that also responded the same way. For example, the 811 genes that were both orthologous and down regulated under drought, represents ~10% of all the rice genes down-regulated following drought stress (all = 8488; Figure 3A; Additional file 2, Table S6). Whilst, these 811 orthologous Arabidopsis genes make up 44% of all the Arabidopsis genes down-regulated following drought stress (all = 1864; Figure 3). Notably, the largest conservation of down-regulated genes were observed for the drought and salt responsive sub-sets, with >40% of Arabidopsis genes showing the same regulation in the respective rice orthologues (Figure 3). In contrast, for the heat and cold responsive sub-sets, <25% of orthologous genes showed a common transcript response between rice and Arabidopsis, despite >60% of the genes in these sub-sets having orthologues in both species.

While examining similarities in the transcriptomic responses between orthologous genes in rice and Arabidopsis, it was observed that some orthologues were responsive in an opposite manner between both species. To determine the extent of this contrasting response, the entire set of rice transcripts significantly up-regulated in each of the abiotic stresses was overlapped with the corresponding Arabidopsis transcripts that were significantly down-regulated for that stress and vice versa (Additional file 1, Figure S2). A small percentage (5%-7%) of rice transcripts that changed in abundance under drought and salt treatment had respective Arabidopsis orthologues that were responsive in an opposite manner (opposite response in R & A; Figure 3). In contrast, an equal or greater percentage of orthologous rice and Arabidopsis genes were found to be responding in an opposite manner in the cold and heat responsive subsets (Figure 3). For example, of the 3062 transcripts up-regulated under cold stress in rice, only 10% had Arabidopsis orthologues that were also up-regulated under cold stress (301; Figure 3), whilst 13% of this up-regulated set (3062) had respective Arabidopsis orthologues that were down-regulated (413; Figure 3). Similarly, under heat stress, the percentage of orthologous genes that were oppositely responding was comparable or greater than the percentage of DEGs that had a common response (Figure 3). For example, the 866 genes that were orthologous and up-regulated genes in both rice and Arabidopsis under heat stress, was smaller than the 969 rice genes that were up-regulated in rice, whilst their Arabidopsis orthologues were down-regulated in response to heat (Figure 3).

**Figure 4:**
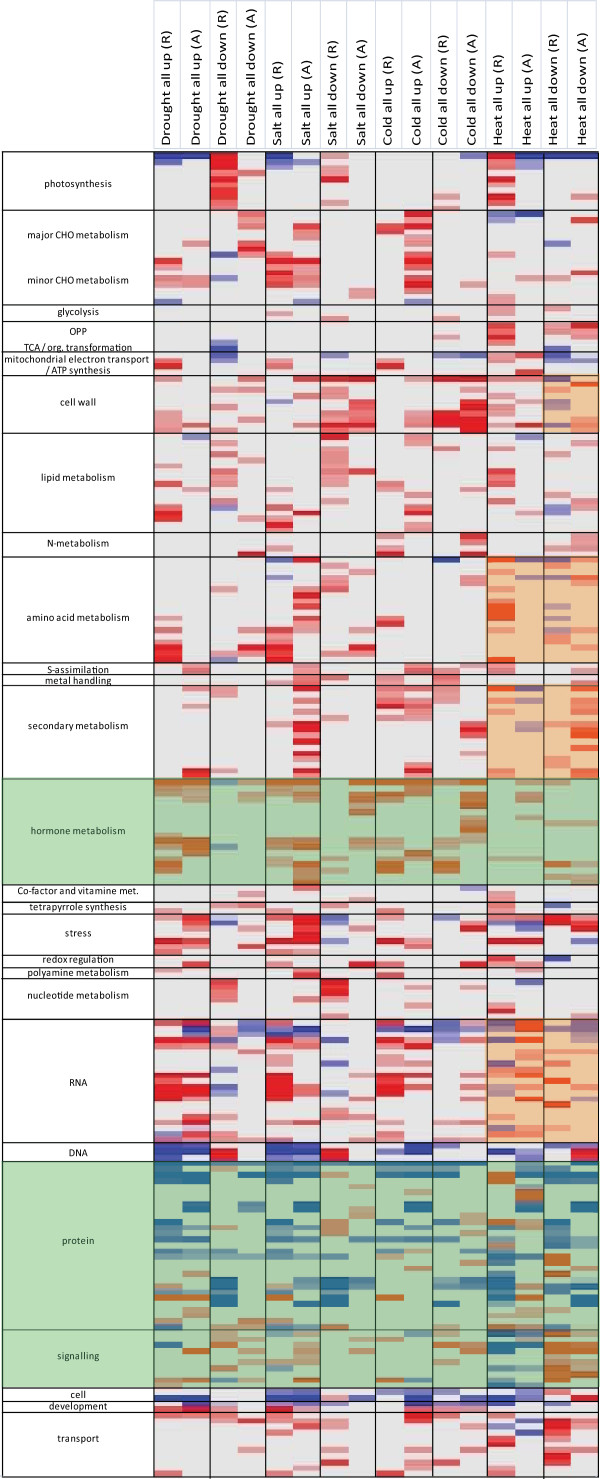
**Pageman analysis of microarray data in response to abiotic stress**. For each abiotic stress experiment in rice and Arabidopsis, the significant fold-changes for the entire set of differentially expressed genes were analysed using the PageMan tool. Statistical analysis of over-represented functional categories was carried out using the Fisher method. Functional categories that did not show significant changes were collapsed for display. Statistical significances are represented by a false colour heat map (up, red; down, blue) where a z-score of 1.96 represents a p-value of 0.05.

In order to examine the overall similarities/differences in the transcriptomic responses (in an orthology independent manner) between Arabidopsis and rice, a comparison of the functions of the DEGs was carried out using the functional over-representation analysis in the Pageman software [33]. This revealed that for some categories such as 'protein' and 'signalling', and to a lesser extent 'hormone metabolism', the over/under-representation patterns of these functional categories were similar (Figure 4, green shade). However, considerable differences in the representation of some functional categories were also evident. This was particularly seen for the genes responding to heat, where the opposite over/under-representation of specific functional categories was observed (Figure 4, orange shading). Specifically, differences in the over/under-representation of 'cell wall, amino acid metabolism, secondary metabolism and RNA functions' were observed in response to heat. Notably for the category of redox functions, it was seen that the abiotic stress responsive subsets in rice often appeared to be over-represented in these functions, whilst this over-representation was not always seen in Arabidopsis. Additionally, for many other comparisons it was observed that Arabidopsis and rice differed in terms of functional representation as a simple comparison of the two columns in each black box displayed more differences than similarities (Figure 4).

**Figure 5:**
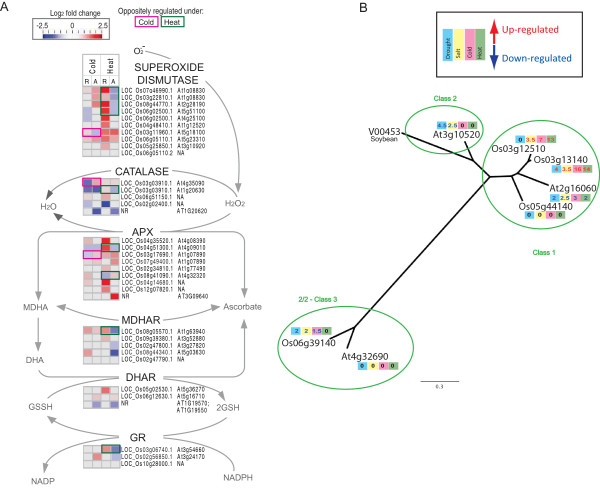
**Visualisation of transcripts showing differential regulation between Arabidopsis and rice**. **A) **Transcripts encoding proteins involved in redox functions, including the ascorbate glutathione cycle components, were visualised on a custom pathway image. The log_2 _fold change is shown for the control vs. treated as a heatmap for rice and Arabidopsis in response to cold, heat conditions. Transcripts showing an opposite response under cold conditions (pink boxes) and heat conditions (green boxes) are indicated. APX-Ascorbate Peroxidase, MDHAR-Monodehydroascrobate Reductase, DHAR-Dehydroascorbate Reductase, GR-Glutathione Reductase. **B) **Phylogenetic analysis of non-symbiotic hemoglobin encoding genes in rice and Arabidopsis. Fold changes in response to each abiotic stress is indicated within the coloured boxes (Drought - Blue, Salt - yellow, Cold - pink, Green - heat) where the colour of the font indicates up-regulation (red) or down-regulation (blue).

In order to examine the genes in these functional groups more closely, individual genes were examined, including the analysis of genes encoding proteins; involved in redox functions, involved in a variety of abiotic stress responses and signalling [34], and non-symbiotic hemoglobins (NS Hbs), involved in nitric oxide removal [35] (Figure 5). Notably, nitric oxide has been reported to be involved in thermo-tolerance [36] and cold acclimation [37], and interacts with ABA and GA signalling in Arabidopsis [38]. A comparison of the DEGs encoding redox functions reveal dramatic changes in transcript abundance in response to heat, with 9 genes showing up-regulation in rice in this pathway, whilst the respective Arabidopsis orthologues were down-regulated (Figure 5A - boxed green). Specifically, it can be seen that 4 genes encoding superoxide dismutases were up-regulated in rice, whilst the respective orthologues in Arabidopsis were down-regulated (Figure 5A). Whilst examining these opposite trends in transcript responses for genes encoding redox functions, the non-symbiotic hemoglobin (NS Hbs) encoding genes also appeared to have this pattern of opposing responses. This was particularly of interest, given that these genes are considered to play an important role in signalling during stress. Thus, the finding that they respond in the opposite manner in rice and Arabidopsis, irrespective of the class of hemoglobin (Figure 5B), suggests that nitric oxide signalling may differ between both species. Notably, other examples of differential over/under-representation of functional groups were also seen for genes involved in amino acids metabolism, secondary metabolism, signalling and transport, which were also seen to respond in the opposite manner under abiotic stress, again, with most evidence of this seen in response to heat stress (Additional file 1, Figure S3).

### Core transcriptomic responses under abiotic stress for rice and Arabidopsis

In order to compare the core abiotic stress responsive genes in rice and Arabidopsis, each of the 4 stress responsive subsets were overlapped based on the transcriptomic response for rice (Additional file 1, Figure S4A) and Arabidopsis (Additional file 1, Figure S4B) independently. For rice, 581 transcripts were down-regulated (Abiotic Down) and 467 transcripts were up-regulated (Abiotic Up) under all 4 abiotic stresses analysed (Additional file 1, Figure S4A). This was smaller for Arabidopsis, with 218 genes up-regulated and 97 genes down-regulated under all 4 abiotic stress conditions (Additional file 1, Figure S4B). For rice, the core Abiotic Up group was found to be significantly (p < 0.01) enriched in transcripts encoding general and amino acid metabolism functions with 27% and 5% of transcripts encoding these functions, compared to 18% and 3% in the genome, respectively. Similarly, the Abiotic Up group in Arabidopsis was found to be significantly enriched in carbohydrate metabolism functions, with 19% of transcripts encoding these functions compared to 9% in the genome. Conserved enrichment was also observed for transcripts encoding transcription factors (TFs), which were significantly over-represented in both the rice and Arabidopsis core Abiotic Up sets, with 23% and 36% of these sets encoding TFs compared to the 16% and 19% that make up all the TFs observed in the rice and Arabidopsis genomes, respectively. Comparison of the down-regulated groups revealed similarities and differences, with the Abiotic Down group in rice and Arabidopsis, both showing enrichment of transcripts encoding kinases (5% vs. 3% in the rice genome and 21% vs. 10% in the Arabidopsis genome). In contrast to the similarities observed thus far, it was interesting to note that there was significant under-representation of transcripts encoding transcription factors (11% vs. 16%) and translation functions (2% vs. 4%), which were only observed in rice, implying divergence in the transcript response for these functional groups.

**Figure 6:**
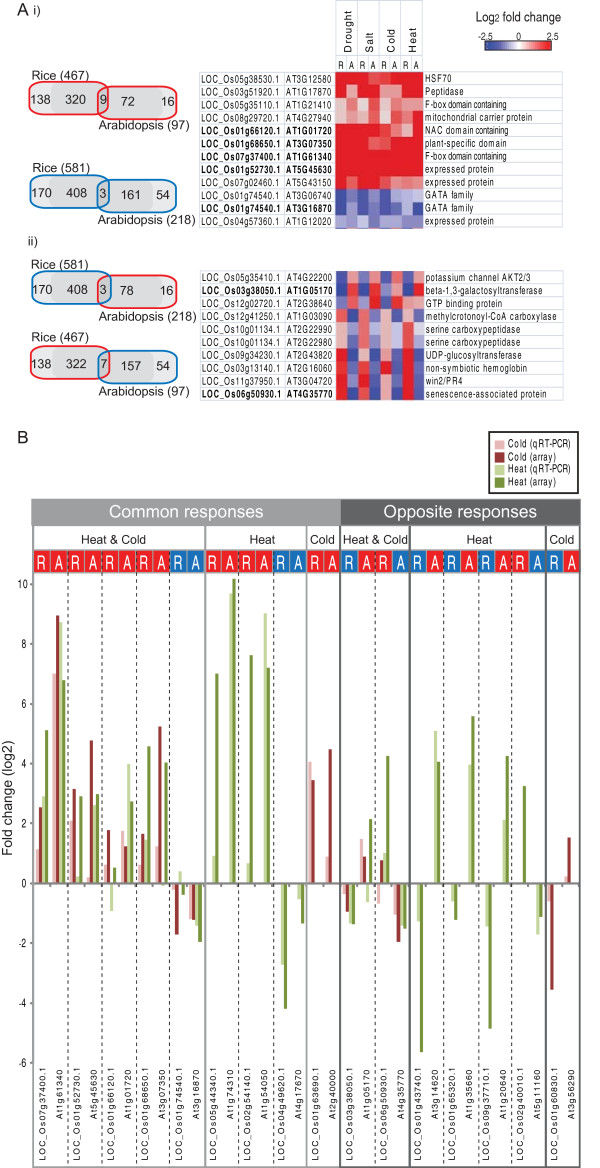
**Core stress responsive gene expression**. The transcripts significantly (p < 0.05, PPDE > 0.96) increasing or decreasing in abundance in rice and Arabidopsis under drought, salt, cold and heat treated plants were overlapped based on their common response (Abiotic core - UP, DOWN, respectively). **A) **Genes which were part of these Abiotic core sets for rice and Arabidopsis were overlapped again in order to determine the number of transcripts with no orthologues (lightest gray shade), with known orthologues (darker gray shade) and the number of transcripts which were orthologous and showing a common (overlap) or opposite (overlap) response across all 4 abiotic stresses. ii) For each of the common and oppositely responding transcripts, the log_2 _fold change is shown for the control vs. treated as a heatmap, where red represents up-regulation and blue represents down-regulation. For Arabidopsis, only a single time point is shown, depending on which time point comparison was significant (Methods section). Genes indicated in bold were also analysed by qRT-PCR (as shown below). **B) **qRT-PCR results showing the log_2 _fold changes of 16 rice genes and 16 Arabidopsis genes in response to cold (dark red) and heat (green) stress. Note that these 16 genes represent orthologous genes in Arabidopsis and rice. The log_2 _fold changes based on the microarray data is also presented for comparison.

For the Abiotic Up/Down sets in rice and Arabidopsis, it was determined how many of these genes had existing orthologues and how many were showing a common response under all 4 abiotic stresses in both species. For the Abiotic Up and Abiotic Down sets in both species, it is evidenced that 72-81% of the transcripts in each set have orthologues, which is not only significantly (p < 0.01) greater than the proportion of orthologues in the rice (37%) and Arabidopsis (61%) genomes, but also higher than the proportions in the single stress comparison sets (Figure 3; Figure 2B). However, similar to the previous observations in this study, the overlap in response of these orthologues is small. Specifically, it can be seen that for the rice and Arabidopsis Abiotic Up sets, only 9 transcripts are orthologous and show the same response at the transcriptomic level under all 4 abiotic stresses in both species (Figure 6Ai). Notably, this included the transcripts encoding the highly stress responsive HSF70, a NAC transcription factor and 2 transcripts encoding F-box proteins, implying a conserved regulatory role for these (Figure 6Ai). This is particularly interesting as F-box proteins are highly conserved in eukaryotes and play a critical role in controlled protein degradation [39]. Unlike the conserved up-regulated set, only 3 transcripts were found to be down-regulated under all 4 abiotic stresses in rice and Arabidopsis, these encoded a single gene of unknown function and a GATA type transcription factor, encoded by a single gene in rice (LOC_Os01g74540.1) and 2 genes in Arabidopsis (At3g06740.1, At3g16870.1) (Figure 6Ai). Again, the presence of a transcription factor implies a conserved regulatory role in both species. Although the roles of some of these protein have been studied such as the heat shock factors and proteases, the role that the two mitochondrial proteins play (At4g27940 and At5g43150) is not known, and the two genes (At5g45630 and At1g61340) have been reported to be regulated by HY5, a bZIP transcription factor known to be involved in regulating light-responsive promoters and chloroplast biogenesis [40], providing direct molecular links between the complex light regulatory network and stress responses [41,42].

In order to examine the transcripts showing opposite response between Arabidopsis and rice, the Abiotic Up and Down sets in rice and Arabidopsis were overlapped (Figure 6Aii). Noticeably, the numbers were very similar to transcripts that changed in a complementary manner outlined above. This analysis revealed that 3 transcripts encoding a potassium ion channel subunit, a beta-galactosyltransferase and a GTP binding protein were all down-regulated under all 4 abiotic stresses in rice, whilst their corresponding Arabidopsis orthologues were up-regulated under all 4 abiotic stresses (Figure 6Aii). Furthermore, 7 transcripts encoding various metabolic functions were found to be up-regulated under all 4 abiotic stresses in rice, whilst the corresponding orthologue in Arabidopsis were down-regulated under all 4 stress conditions (Figure 6Aii). In addition to the NS Hbs discussed above (Figure 5B), a mitochondrial protein involved in leucine degradation (At1g03090) and a protein induced by phosphate starvation (At4g35770) were also notable. For the other five genes however, no information was available.

### qRT-PCR validation of responses to cold and heat stress

In order to independently validate the transcriptomic responses of the genes found to be responsive to heat and cold stress, 16 rice genes and their respective Arabidopsis orthologues (32 genes in total) were analysed by qRT-PCR. It can be seen that genes showing the core common responses to abiotic stress showed responses comparable to the observed responses using microarrays (Figure 6B). These genes also included a heat shock factor (marker for heat stress) and other stress responsive genes (Figure 6B). Similarly, the genes found to be oppositely responsive were also responding in a similar manner to the microarray data, including the gene encoding SEN1, which was found to be up-regulated in rice, whilst the Arabidopsis orthologue of this gene seen to be down-regulated (Figure 6B). The changes in transcript abundance in response to cold and heat stress were consistent between the qRT-PCR data obtained directly experimentally in this study to that of the published microarray data for these stresses. Overall, the transcript abundance in response to cold and heat stress, showed a conserved pattern between the independently derived qRT-PCR data (from this study) and responses based on the microarray data. In addition, to confirm the validity and effectiveness of the stress treatments carried out (resulting in the microarray data), it was useful to examine the response of marker genes such as HSFs for heat and AP-2 transcription factors for cold stress (Additional file 1, Figure S5A and B). Phylogenetic analysis of a subset of known heat responsive HSFs and known cold responsive AP-2 TFs showed that these genes were not only closely related but also showed the expected up-regulation responses to heat and cold stress. In addition, this phylogenetic analysis was also carried out for the related genes of SEN1 in rice and Arabidopsis (Additional file 1, Figure S5C). As expected, it was seen that despite the close relation between SEN1 in rice and Arabidopsis, there was difference in expression observed with up-regulation observed in rice and down-regulation observed in Arabidopsis (Figure 6B; Additional file 1, Figure S5C). These findings indicate that despite the variety of sources from which the microarray data was derived from, the normalisation and analysis processes are robust and the trends observed can be reproduced independently. Thus, the differences in responses cannot be attributed to experimental variation alone.

### Regulation of transcription factors under abiotic stress for rice and Arabidopsis

**Figure 7:**
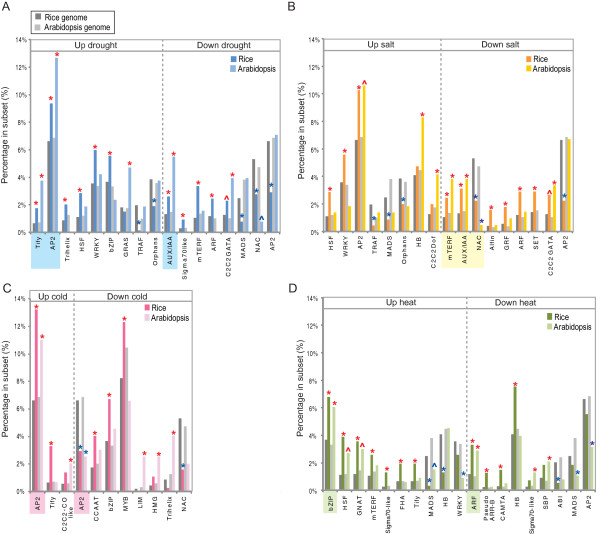
**Analysis of changes in transcript abundance for genes encoding transcription factors**. All the genes encoding transcription factors were collated for Arabidopsis and rice and the distribution of these transcription factor families changing in abundance within the total stress responsive sets were examined for **A) **drought (blue), **B) **salt (yellow), **C) **cold (pink) and **D) **heat (green). The distribution of transcripts encoding transcription factors in each family, within is stress responsive subset (e.g. Up drought) was compared to the percentage present in the respective genome. In this way, over/under-represented transcription factor families within each subset could be determined for Arabidopsis and rice separately. Over- or under-representation is indicated by red or blue asterisk respectively (at p < 0.01). Note that some transcription factor families were statistically significant at p < 0.025, these are indicated by ^. The % breakdown of each family within each subset is shown next to the genome (lighter shades - Arabidopsis, darker shades (rice).

The above analysis indicates that the transcriptional response to abiotic stress differs significantly in terms of gene orthology and function between Arabidopsis and rice. This suggests that the regulation of these responses may also have changed and thus the transcription factors underlying these responses and the *cis*-elements that define transcript abundance were analysed to determine if they also displayed significant differences. In both Arabidopsis and rice, a large number of genes encode transcription factors (TFs) [43]. Defining orthology for TFs removes many TFs for both species, as they are grouped into families based on structural features or small domains. Therefore, to compare the transcript abundance of genes encoding TFs, a comparison was made based on family classification. In this way, the different sizes of the families in both species would be taken into account. Only significantly (z-score, p < 0.01) over/under represented TF families in each subset are shown in Figure 7, however if one family is significantly over- or under- represented at p < 0.01 in rice or Arabidopsis, and it is significant at p < 0.02 in the other species, it was indicated with a + in the latter (Additional file 2, Table S7). Overall, the most conserved changes between rice and Arabidopsis were observed with the AP2 family of TFs, with comparable changes observed in the drought (up), salt (up) and cold (up, down) responsive sub-sets (Figure 7). However, even for this family, different responses were observed in the drought and salt sub-sets for down-regulated genes (Figure 7). Also, in these down-regulated drought and salt responsive subsets, the AUX and NAC family of TFs were found to be significantly over-represented (Figure 7). A number of other families were conserved in response to the single stress treatments such as Tify (drought up), mTERF (salt down), bZIP (heat up), ARF (heat down) and HSF (heat up, p < 0.02 for Arabidopsis) in Arabidopsis and rice. However, under the four conditions examined several TF families are specifically over-represented in rice alone such as the trihelix, WRKY, and bZIP families, whilst the GRAS family was over-represented in Arabidopsis. Likewise in many instances, genes encoding specific TFs were under-represented, such as HB in the heat responsive set (up) in Arabidopsis and WRKY in rice (Figure 7). Thus, despite the conserved enrichment for some TF families (e.g. AP2 in response to cold stress), this pattern was not always consistently observed across both species. The observed differential expression and differential representation of transcription factors between rice and Arabidopsis complements the observed divergence in the transcriptomic responses following abiotic stresses between both species.

Given the significant difference in genome size between the rice and Arabidopsis, it is important to consider the potential effect of duplication and pseudo-functionalisation on the in the results observed. As with all the comparisons in this study, each gene family was compared to the respective genome before further comparisons were made across species, eliminating the bias effect of the genome size. A good example of this is for the AP2 TF family, it can be seen that despite the difference in genome sizes, the proportions of AP2 TFs were conserved, making up 6.64% and 6.86% of all TFs in rice and Arabidopsis, respectively. Similarly, HSFs make up 1.12% and 1.2% of all TFs in rice and Arabidopsis, indicating a conserved representation of these across both genomes. Therefore the conserved over-representation of AP2 TFs and HSFs in the sets of genes up regulated in cold and heat stress respectively represents an unbiased, conserved response. Furthermore, when sub-groups of AP2 TFs (DREBs) and HSFs were phlyogenetically analysed, the orthology between the genes in each of these subsets were clearly seen, as well as a conserved pattern of up-regulation (Additional file 1, Figure S5B and C) indicating conservation, both at the gene and expression levels for these families. In contrast, it can be seen that despite similarity in TF family size e.g. for the WRKY family (3.56% and 3.38% of all TFs in rice and Arabidopsis, respectively), this family was only found to be over-represented in the drought and salt responsive sets in rice and not Arabidopsis, revealing species specific differences in the response of these genes.

### Promoter Analysis of transcriptome of Rice and Arabidopsis

**Figure 8:**
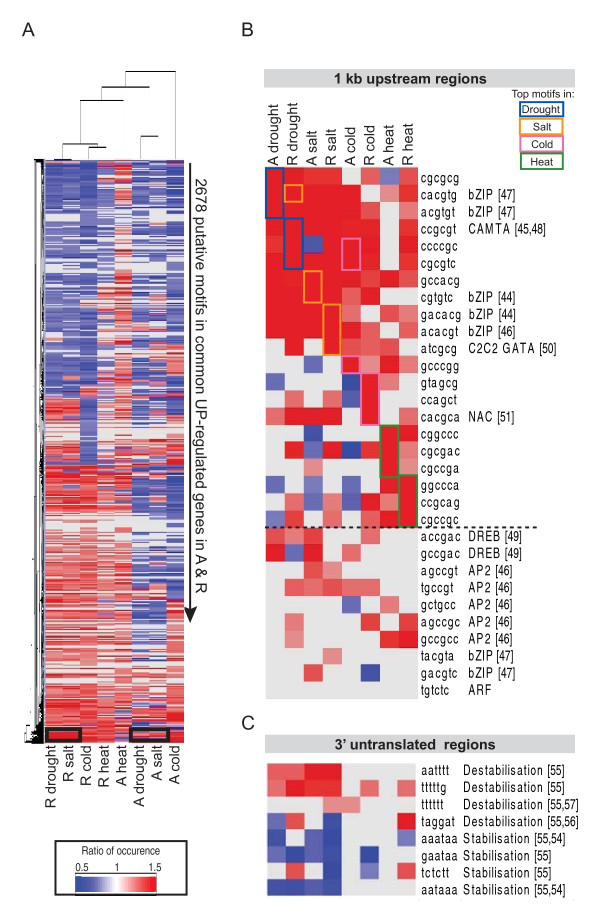
**Analysis of putative motifs for core orthologous genes showing common response under abiotic stress**. **A) **The occurrence of all possible 6-mers was calculated in each of the core orthologous up-regulated subsets for rice and Arabidopsis. The occurrence of each motif in a subset was made relative to the occurrence of that motif in all promoters in the respective genomes. Heat map intensity represents over (red) or under (blue) representation of putative motifs compared to the genome. From the 4,096 possible 6-mers, only the putative motifs present 20% more or less often than the percentage presence in the genome were visualised e.g. a value of 1.2 indicates that this motif in the respective subset occurs 20% more than the occurrence of this motif in the genome, whilst a value of 0.8 indicates that this motifs occurs 20% less than the percentage occurrence in the genome. Examples of conserved over-represented motifs within different stress subsets are indicated in the black boxes. **B) **The top 3 over-represented motifs from each of the up-regulated subsets are indicated on the heatmap by the coloured boxes. Note that motifs over-represented in the UP and DOWN regulated sets are indicated by an asterisk *. For the motifs representing known TF binding sites, the respective TF is annotated; the number in brackets indicates the associated reference. **C) **Examination of over/under-represented known 3'UTR motifs in each subset. Note that the number in brackets indicates the associated reference.

Previous studies and the analysis so far have focussed on expression levels, with this study focussing on similarities and differences in the expression of orthologous genes. A gene is defined as orthologous when there is commonality in the function of the encoded protein, and thereby similarity at the level of the protein sequence as well. Considering this, it was asked whether the genes that were both orthologous and showing a common response were also regulated in the same manner i.e. share commonality in promoter/transcriptional regulation. To answer this, putative *cis*-acting regulatory elements (CAREs) were examined in each of the orthologous sub-sets regulated in the same way. Specifically, all 4,096 possible 6-mers were counted within the 1 kb upstream regions for all genes in the rice genome (TIGR6) and Arabidopsis (TAIR9) genome, as well as for each sub-set. The occurrence of the putative motifs in each set of DEGs was made relative to the occurrence in all the genes in each respective genome. In this way, it could be seen whether a sub-set of genes contained a significantly greater or smaller percentage of putative motifs in the promoters of these genes compared to the percentage occurrence in the promoters of all the genes in the genome (denoted as a value of 1; Figure 8). Given that a large number of motif counts were generated for each subset and a large number were significant (p < 0.01), a cut-off of 20% was set for short-listing the putative motifs. That is, the final set of putative motifs showed the motifs that were significant AND present 20% more/less than the percentage occurrence of that putative motif in the genome.

Ratios of the putative motif counts to the genome was hierarchically clustered, where the bright red represents motifs that occurred at least 50% more often than the percentage occurrence in the genome (i.e. ratio of 1.5 or more) and the darkest blue represents the opposite under-representation (Figure 8A). Furthermore, the putative motif occurrence ratios were not only clustered based on the ratios to the genome (rows) but also clustered on the sub-sets as a whole i.e. clustered on columns in order to examine similarities the overall pattern of motif occurrences between species and subsets (Figure 8A). It was immediately evident that the pattern of occurrence of putative CAREs differs between species as Arabidopsis and rice forms two distinct branches (Figure 8A). There appears to be more similarity within a species in response to a number of stresses rather than between species in response to the same stress. This was especially seen for Arabidopsis, where the DEGs responding to drought and salt, and cold to a lesser extent, displayed similar patterns of CAREs present in their promoters (Figure 8A; Additional file 1, Figure S6). In contrast, the occurrence of CAREs in rice appear to be distinct for each stress, this may be a reflection of the larger number of responses in rice for drought and salt (Figure 3). Again, in response to heat, there appears to be a distinction in terms of motif occurrence for the rice DEGs (Figure 8A).

Looking for common elements between species it was noticeable that there were common elements in both the drought and salt response in Arabidopsis and rice (Figure 8A, black boxes). The 3 significant (p < 0.01) and most over-represented motifs ("top ranked") are shown in Figure 8B. Searches for these putative motifs across relevant publications ([44-51] in Figure 8) revealed that 5 of the top over-represented motifs appear to be ABREs and are targets of bZIP transcription factors [47-50]. Noticeably, the AP2 transcription factor family (drought and salt up) and Auxin and NAC (drought and salt down) display conserved responses in these treatments (Figure 7), suggesting that they may bind these motifs. This is also supported by the finding that the motif, "cacgtg" (ranked 2; Figure 8B) appears to be conserved and over-represented in the genes up-regulated under drought and salt in Arabidopsis and rice and this motifs is similar to the recognised NAC binding site [52]. Interestingly, a similar observation was also made for the putative motifs over-represented under cold conditions in both species, where GC-rich motifs, characteristic of AP2 TF binding sites were over-represented in the cold responsive transcripts (Figure 8Bii) [53]. Again, divergence in the heat response was further supported by the finding that the top three elements over-represented in heat responsive genes (green boxes) do not overlap with the top three putative motifs from the DEGs under other stresses (Figure 8B).

Lastly, given the large number of down-regulated genes observed in drought and stress conditions, we examined the over-representation of putative motifs in the 3'UTRs for motifs associated with mRNA degradation. Ideally, this would be compared directly as done for the promoters, identifying the most over-represented motifs, however less than 10% of all rice genes have sequence information available for the 3'UTRs, thus it was only feasible to examine previously identified/implicated motifs associated with mRNA degradation. Stabilisation and destabilisation motifs previously identified [54-57] were examined and interestingly for the genes in the drought and salt responsive sub-sets, a significant over-representation of destabilisation and significant under-representation of stabilisation motifs were observed, corresponding with the larger number of down-regulated genes observed in rice and Arabidopsis following drought and salt stress.

## Discussion

A major role of model organisms is to facilitate investigation and gain insights into biological mechanisms that can be applied to other organisms. A good example is how research on Arabidopsis impacts on research in the biomedical field [58], despite the ~1 billion years since divergence. More commonly, research using model species is usually used in comparisons within phylogenetic boundaries, i.e. plant, animal or fungi. Arabidopsis and rice are the forefront plant dicot and monocot models, respectively. A variety of comparisons between these species have been carried out from the genome [59], proteome [60] and metabolome levels [61] revealing insights into conserved and distinct features and responses. At a transcriptome level, a variety of comparisons have also been carried out with a variety of gene families (see introduction for examples). In this study, a comparison of organ and abiotic stress responses was carried out at the global transcriptome level. Given that abiotic stress responses were compared between rice and Arabidopsis, it is important to point out that each of these species has evolved different mechanisms or tolerances optimising their survival in their native climates. This is particularly relevant in terms of optimal climatic conditions, where Arabidopsis is grown in a temperate climate (22°C) [62], whilst optimal growth conditions for rice is in a tropical climate (28-30°C) [63]. Therefore, any results derived from comparisons to extreme temperature stress would reveal not only the response to the temperature but also incorporate the natural responses to the respective temperature. The comparisons in this study were done to define similarities and differences at a global level for the first time. While similarities point to conserved processes and provide targets to dissect basic core processes, differences provide opportunities to identify different approaches that have arisen in evolution. Note that differences also represent the divergent responses to the conditions applied, not just a reflection of the basal transcriptome, and thus have the potential to reveal the diversity of responses to altering conditions. While analysing the differences between closely related varieties of a species, such as in Arabidopsis, in which whole genome approaches can now be applied to tens or even hundreds of varieties, has the potential to reveal the diversity in responses within essentially a very similar set of genes, analysis between different species will reveal alternative responses.

When carrying out comparisons between species, the typical assumption is that similar genes (defined as orthologues) are likely to have similar responses. When analysing both transcript response and orthology, it is important to consider the stringency in the definition of orthologues. Previous studies, may have over-estimated the proportion of rice genes that have Arabidopsis orthologues and vice versa, however, these estimates were most likely based on the sequence of genes that had been determined and over-estimated. The data presented here is conservative, in that the definition of orthology is based on the Inparanoid data - which could be considered a gold standard. However, even using functional based definitions of gene function (e.g. Pageman FUNCATs) the responses are still observed to be divergent for certain functional groups as a whole. Thus, irrespective of the strict definition of orthology, or even same the functional categorisation, the responses between Arabidopsis and rice appear to be divergent for specific groups of genes, particularly in response to abiotic stress.

This study revealed that the transcriptional network and underlying *cis *and *trans *regulatory factors of rice and Arabidopsis differs significantly in many aspects. Only by multi-dimensional analysis of gene expression across a multitude of microarray studies in both species, was it possible to get an idea of the transcriptomic flexibility i.e. the proportion of genes showing tissue specific or differential expression under various abiotic stress conditions. Combining expression with orthology revealed the danger in assuming orthology also reflects similarity in transcriptomic or even proteomic response. Firstly, the specific transcriptomes of rice seeds, flower, leaf and root was seen to be distinct to that of Arabidopsis, notably complying with findings from other transcriptomic studies [32,64]. This lack of orthology between these "organ specific" genes indicates that the observed morphological differences between rice and Arabidopsis organs are also correlated with distinct gene expression and although correlation is not cause, it does present a possible avenue by which distinct morphology can be obtained. While the overlap in organ specific expression between Arabidopsis and rice is low at the level of gene orthology, for roots the overlap in function for many categories is much higher. Thus, despite differences in roots between monocots and dicots [65], it appears that at a functional level, the organ specific transcript profiles are more conserved compared to flower, seed and leaf.

In contrast, it was observed that a significantly larger proportion of genes with orthologues were present in each of the abiotic stress responsive sets compared to the percentage of orthologues in the whole genome level. This suggests that although distinct genes may explain morphological differences between species, it may not be the reason for differential tolerances to abiotic stresses. Interestingly, the proportions of genes showing comparable, opposite and unchanging responses to abiotic stresses differed between rice and Arabidopsis. These represent particularly important findings, given that most studies tend to focus on similarities between plants, especially when considering orthologous genes. In rice, there were more than 4 times as many DEGs following drought and salt stress compared to the number of DEGs following these stresses in Arabidopsis, and this cannot only be accounted for by differences in the gene coding content of the respective genomes, as the number of differentially expressed genes in response to heat and cold for both species was approximately the same. In addition, this is also not due to species specific rice gene expression as there is in fact a larger percentage of known Arabidopsis orthologues for the rice genes changing under drought and salt stress (> 50%) compared to the genome percentage (~31% Arabidopsis orthologues). Noticeably, despite differences in the number of DEGs, it was evidenced that the number of DEGs showing opposite responses was much lower than the number of DEGs showing complementary changes, indicating that the response to drought or salt stress is more conserved. Closer examination of the orthologous genes showing common expression responses revealed the conserved down-regulation of translation functions and up-regulation of membrane transport functions in both Arabidopsis and rice. This suggests that under these conditions, energy demanding process such as translation are down-regulated, whilst the change in water content under drought or salt stress affects membrane fluidity in a conserved manner.

Interestingly, the number of DEGs under heat and cold stress were found to be comparable across rice and Arabidopsis. However, it was surprising to find that equal or more orthologous genes responded in the opposite manner as in those responding in a similar manner, providing novel evidence for a divergence in the regulation of these genes in response to temperature extremities. Specifically, an overall up-regulation of rice genes was observed compared to the respective orthologous genes in Arabidopsis following cold or heat stress. This suggests that the regulation of gene expression in response to these temperature extremities has diverged, with genes involved in secondary metabolism, amino acid degradation and redox metabolism being up-regulated under these conditions in rice, whilst the Arabidopsis orthologues remained unchanging or oppositely regulated. Overall, it is important to note that the number of DEGs observed in response to abiotic stress between Arabidopsis and rice cannot only be attributed to the differences in the size of gene families or duplication of genes. A specific example of this principle was seen for the non-symbiotic haemoglobin encoding genes, where the transcripts for all gene family members responded in opposite manners.

Given the general down-regulatory trend observed for the transcripts encoding redox pathway components in Arabidopsis following heat treatment, it was of interest to consider the regulatory mechanisms that may be controlling this down-regulation. Typically, transcriptional control is examined as the main mechanism regulating transcript abundance, however, given the observed down-regulation, a role for mRNA degradation was also considered. A previous study examined the global mRNA degradation rates for Arabidopsis, following transcriptional inhibition [55]. Brief examination of the mRNA half-lives of the genes encoding redox pathway components (as in the Arabidopsis genes in Figure 4) revealed remarkably high mRNA half-lives (> 10 h) for several of these genes, including 2 genes encoding catalases (AT4G35090, AT1G20630), a gene encoding DHAR (AT5G16710), 2 genes encoding dismutases (AT4G25100, AT3G10920), a gene encoding GR (AT3G54660) and the gene encoding a non-symbiotic hemoglobin (AT2G16060). mRNA half-lives of >10 h indicates a high level of stability of these mRNAs, thus it was surprising to note the significant down-regulation of these within only 3 h of heat treatment in Arabidopsis. Similarly, a large down-regulation of transcripts encoding translation functions is seen to occur in both Arabidopsis and rice following drought or salt treatment, again within only 3 h of stress treatment. The observed rapid down-regulation of these transcripts in Arabidopsis was somewhat surprising, given the expected high level of stability of these transcripts [55] and may be explained by a mechanism of active degradation. A previous study in yeast revealed that in response to changes in oxygen availability, there is active degradation of specific transcripts that occurs faster than the steady state decay rates [66]. Considering this, it is possible that part of the response mechanism to different abiotic stresses is the active degradation of specific transcripts, e.g. the possible active degradation of transcripts encoding redox pathway components following heat treatment in Arabidopsis.

In terms of transcriptional regulatory processes, it also appears that there were more differences than similarities between rice and Arabidopsis. Global comparison of all over-represented, putative *cis*-acting sites within the 1 kb upstream regions of co-expressed orthologous genes revealed some unexpected findings. Specifically, it was observed that even when similar expression patterns were observed under abiotic stress for orthologous genes in both species, the regulatory mechanisms that drive these responses appeared to differ, with some exceptions. Overall, these findings indicate that even when two plants have similar genes (orthologues) in their genome and even if these genes show similarities in their transcriptomic responses, it is possible that the factors regulating this gene expression can differ. To consider these factors in both species, the expression of TFs was considered. Notably, the CAREs and TF families of AP2 and NAC were found to be enriched in both species in response to several abiotic stresses, complying with the previously established conserved regulatory role for these families in both Arabidopsis and rice [53]. Thus, the outcome of the *in silico *analysis in this study was strengthened by the support of previous experimental observation. Notably, although some NAC TFs responded in the same manner between Arabidopsis and rice under certain abiotic stresses, it has also been suggested that NAC TFs may have additional roles in rice [53]. Other groups of TFs also enriched in the response of both species to specific stresses included the Auxin TFs in response to drought and salt, and bZIP, HSF and GNAT in response to heat. With the exception of the cold responsive set, overall, more families of TFs appeared to be affected in rice, compared to Arabidopsis. This suggests that in rice, the regulatory network in response to abiotic stress is more diverse and that TFs have addition roles in the stress response.

In addition to transcriptional regulation, the changes observed in transcript abundance observed are also the result of post-transcriptional regulation. Interestingly, a brief search of the genes encoding the oppositely regulated transcripts in Arabidopsis revealed some examples of genes with known to be regulated at the post-transcriptional level. For example, a recent study of the regulation of SEN1 (At4g35770) revealed that the transcript level for this gene as well as two other genes; a gene encoding a leucine zipper TF (At2g22430) and an gene with unknown function (At3g26740) are regulated by a controlled mRNA degradation mechanism [67]. Targets of this controlled mRNA degradation pathway, including the aforementioned genes, are characterised by the presence of the downstream (DST) motif in their 3'UTRs and this motif is associated with rapid degradation of these transcripts [67]. Notably, in Arabidopsis, all 3 of the aforementioned genes are down-regulated in response to one or more abiotic stresses whilst the rice orthologues of these genes are up-regulated or unchanged in abundance for that stress. In addition, a brief search for the DST motif in the rice orthologues of these genes revealed that the DST motif was not present in these 3'UTRs, providing an example that indicates potential divergence in post-transcriptional regulation between Arabidopsis and rice.

The differences in the transcriptome response of Arabidopsis and rice provides opportunities to identify species specific responses and thus broaden the possibilities of transferring traits from model to crop species. In this respect, the orthologous genes that significantly changed in opposite directions warrant further investigations. Notably, the transcripts of many genes in these various sub-sets encode proteins involved in stress defence and are classified as such. The control and regulation of genes involved in various processes classified as redox, biotic stress and secondary metabolism, which are all intimately associated with stress responses, will likely reveal species specific regulation and response that may offer new insights for translational biology. In particular, the fact that a non-symbiotic hemoglobin gene is regulated in the opposite manner in rice (up) compared to Arabidopsis (down) in all 4 stresses merits investigations into nitric oxide signalling differences that may exist between both plants in response to abiotic stress [68]. In addition, the finding that transcripts encoding components involved in redox functions also displayed opposite trends under heat stress, suggests that differences in reactive oxygen species or reactive nitrogen species related functions may exist between both species. Although there are reports where the expression of heterologous genes in crop species confers resistance to a stress [23], this is often accompanied by growth penalties [66]. One possible reason for the observed growth penalties is the different interaction and/or regulatory environments of genes (or more correctly the proteins they encode) in different species. Thus, although reactive oxygen or nitrogen species are likely to play signalling roles in both Arabidopsis and rice under a variety of conditions, especially stress, the response to these signals may differ significantly.

## Conclusions

Large scale systems biology projects are well advanced to define the function of all genes in Arabidopsis and to understand genome to phenome relationships under various environmental conditions [69,70]. However, in order to fully exploit the power of Arabidopsis as a model, it is as important to know what is common and different with other plants, and also what responses in other plants may be unique, in that they are not observed in Arabidopsis. Given the different native temperature growth conditions and the ~140 million years of divergence between Arabidopsis and rice, it is vital to understand the level of comparability between these species. Differences in the transcriptomic response to heat stress was particularly of interest, as these responsive genes may represents key genes for translational biology studies aiming to increase heat tolerance in other plants, which is a growing area of research given the observed increase in global warming. Overall, the results of the analysis presented here reveal for the first time at the global level, that there is not a single relationship between genome, in terms of gene orthology, and phenome across species. Rather, there are likely to be a number of dynamic combinations of a genome or transcriptome to produce a phenome, and these combinations will differ between plants depending on both the environment and evolutionary distance that allows divergence. This cautions against assumptions that orthology equals similarity in cellular responses. It also provides a rational basis for the selection of candidate genes for translational research. Specifically, via the selection of genes that respond in a similar manner or transcription factors that regulate genes in a similar manner between species. Furthermore, the genes responding in an opposite manner do not necessarily represent barriers to translation; rather they represent opportunities to equip plants with novel solutions to environmental stimuli.

## Methods

### Publically available rice and Arabidopsis microarrays

To compile the entire publically available Affymetrix rice microarray (as at 1^st ^August 2009), all experiments containing CEL files were downloaded from the Gene Expression Omnibus within the National Centre for BiotechnoIogy Information database or from the MIAME ArrayExpress database http://www.ebi.ac.uk/arrayexpress/. The GSE or EXP numbers for the respective rice studies are shown in Table 1. The rice array was defined as the 57,302 probesets, thus the 81 probesets designed for the bacterial/phage controls were not included. There was a total of 366 microarrays, representing 129 tissues/conditions, with a minimum of 2 biological replicates for rice. The 129 included 48 developmental tissues, 77 samples within abiotic and biotic stress experiments and 11 samples within hormone treatment experiments. The abiotic stress experiments involved single treatments with cold, salt, drought (GSE6901) and heat (GSE14275). To carry out parallel analysis for developmental conditions in Arabidopsis, the Arabidopsis developmental dataset consisting of 237 microarrays representing 79 developmental stages was downloaded as CEL files (E-AFMX-9; E-TABM-17). Also, to carry out parallel analysis of abiotic stresses in Arabidopsis, the microarray data from the 0.5 h, 1 h and 3 h abiotic stress treated (cold - GSE5621, salt - GSE5623, drought - GSE5624, heat - GSE5628) and respective control samples (E-GOED-5620) was downloaded. Thus, a total of 627 microarrays were analysed within this study, enabling large scale parallel comparison between rice and Arabidopsis at a transcriptomic level during development and under four abiotic stresses. Note that for Arabidopsis, the abiotic stress experiments involved shoots from 18-day old seedlings, whilst in rice these stress treatments were carried out on 7-day old seedlings for the drought, salt and cold stress conditions [39] and 14-day old seedlings for the heat stress experiment [71] (Table 1).

### Microarray analyses

All raw intensity CEL files were imported into Avadis 4.3 (Strand Genomics, India) and the standard MAS5.0 normalisation was first carried out in order to determine present/absent/marginal calls for each probeset. All probesets that encoded bacterial genes were excluded, leaving a global rice set consisting of 57,302 probesets and a global Arabidopsis set of 22,710. Probesets that were called present in two or more replicates were considered to be expressed and used for further analysis. All of the present/absent data for all 366 microarrays were compiled and it was determined which probesets were present in all 129 tissues/conditions, defined as "Always expressed" and which were present in none of the microarrays, defined as "Never expressed" on microarrays. Furthermore, a probeset was considered to be transiently or "Specifically expressed" if it was present in only one sample i.e. present in at least 2 replicates of one sample and less than 2 replicates in all other samples. In this way, developmental stage/tissue specific probesets were identified for both rice and Arabidopsis. However, this method only included single stage/tissue specific probesets, thus this was expanded further to include probesets present in one or more developmental stage for that tissue e.g. at one or more stage of inflorescence (and less than two replicates for all other tissues).

### Differential expression analyses

In order to analyse the rice microarray data from the abiotic stress experiments, differential expression was carried out. Firstly, a present set for each experiment was determined i.e. present in >/= 2 replicates in the control and/or treated samples and only these were included for further analysis. For each stress experiment, the GC-RMA normalised data (control and stress treated) were used as the input set for the differential expression analysis. This was carried out using the Cyber-T method, which implements a Bayesian method for determination of probesets showing significant changes in transcript abundance. The PPDE method within Cyber-T was used for false discovery rate calculation. All input criteria were set according to Cyber-T recommendations applicable for each experimental set. A probeset was defined as significantly changing at p < 0.05, with a PPDE of >0.96 (false discovery rate). These cut-offs and this Bayesian method of differential expression has been verified and has been used in previous microarray studies [72,73]. For each abiotic stress experiment in rice, single comparisons were involved i.e. treated vs. control. For Arabidopsis, these experiments were carried out as a time course, however only the 0.5 h, 1 h and 3 h post-treatment microarrays were considered for analysis as this was considered to be a more parallel reflection of the stress treatments in rice and also, it was the only time points consistent across all 4 stress experiments i.e. heat stress was not sampled after 3 h in Arabidopsis. In this way 4 comparisons for rice; and 12 comparisons for Arabidopsis; abiotic stress vs. control were carried out. For Arabidopsis, the differential expression analysis involved a comparison for each time point, within each stress experiment and the differentially expressed list was generated on the criteria that a probeset had to be changing (up/down) at one or more time points in the same direction (up/down) with no significant changes in the opposite direction.

### Functional annotation and statistical analysis

For all the transcripts represented on the rice microarray, the function of the encoded proteins was analysed, where functional annotation was available. For each probeset, the GO annotation and transcript assignments from Affymetrix was retrieved. The National Science Foundation rice microarray database was used to match rice probeset identifiers to The Integrated Genome Resource (TIGR) identifiers for rice. Similarly, The Arabidopsis Information Resource (TAIR) database was used to match Arabidopsis probesets identifiers to Arabidopsis Genome Identifiers (AGI) as At numbers. For all the rice transcripts matched to a single TIGR identifier, the rice TIGR database (Yuan et al) was used to determine putative protein functions. Note that only ~80% of all probesets had annotated TIGR identifiers. To analyse transcripts based on the broad function of the encoded protein, the FUNctional CATalogue (FUNCAT) for rice based on the Australian National University Gene-bins database was used for the whole genome set. To improve and add to this, two FUNCATs; transcription factors and kinases were independently added. The list of transcription factors was based one or more of the following sources; DRTF [74], RiceTFDB [75], and Caldana et al., 2007 [31]. Kinases were annotated based on the rice kinase database [76].

In order to determine if there was a statistically significant over or under-representation of a particular FUNCAT within a subset compared to the genome, a z-score analysis was carried out based on the difference between the two proportions, given that the sample sizes and frequency of each FUNCAT is known:

$$z=\frac{{\hat{\pi }}_{1}-{\hat{\pi }}_{2}}{\sqrt{\hat{\pi }(1-\hat{\pi })(\frac{1}{n_{1}}+\frac{1}{n_{2}})}}$$

A cumulative standard normal Table was used to match the z-score and based on this, the P values were determined. The same method was used for transcription factors where the proportion of transcription factor families within a subset was compared against the full list of transcription factors.

### Pageman analysis

In order to analyse the rice microarray data from the abiotic stress experiments, differential PageMan [33] and analyses were carried out using a reduced set of unique probesets representing the differentially expressed genes. For the Pageman analysis, Fisher's test for ORA (over-representation analysis) analysis was carried out in Pageman in order to determine statistically significant over/under representation of genes classified into specific BINS. Given that Pageman does not allow visualisation of data from more than species at once, the raw data for rice and Arabidopsis was exported, matched and visualised in parallel using Partek Genomics Suite (version 6.5) to produce the output seen in Figure 2D and 4.

### Analysis of orthologues

The InParanoid: Eukaryotic Ortholog Groups database (version 7.0) was used to analyse all orthologues between rice and Arabidopsis [30]. The orthologous group file was downloaded for the whole-genome comparison of rice versus Arabidopsis. This produced information for orthologues identified by TIGR identifiers for rice and AGIs for Arabidopsis.

### Phylogeny analysis of genes

Using the protein sequences for the AP2, HSF, SEN1 and non-symbiotic hemoglobin encoding genes in rice and Arabidopsis, multiple sequence alignments were carried out using MAFFT [77] and visualized using Multiple align show http://www.bioinformatics.org/sms/multi_align.html. The program IQPNNI [78] was used to reconstruct a maximum likelihood phylogeny assuming the Whelan and Goldman model [79]. Phylogenetic trees were finally visualized using the program Geneious http://www.geneious.com. For the non-symbiotic haemoglobin phylogenetic tree in Figure 6, the sequence for a non-symbiotic hemoglobin-encoding gene in soybean was also included as an outside comparison.

### Stress treatments, tissue collection and RNA isolation

In order to analyse the response to heat and cold stress for Arabidopsis and rice independently from the microarray data, expression analysis was carried out for the genes shown in Figure 6. For Arabidopsis, Col-0 seeds were grown on MS liquid media at 22°C for 2-weeks. Two hours into the light, the 2-week old seedlings were transferred to 4°C for cold stress and 38 C for heat stress (as carried out by Kilian et al., 2007 [80]) for 3 h, whilst the controls remained at a constant temperature of 22°C. For rice, wild type cv. Amaroo seeds were grown for 2 weeks at 30°C. Two hours into the light, the 2-week old seedlings were transferred to 4°C for cold stress (as carried out by Jain et al., 2008 [39]) and 42°C for heat stress (as carried out by Han et al., 2009 [71]) for 3 h, whilst the controls remained at a constant temperature of 30°C. All tissue samples were grown and collected using three biological replicates for each control and treatment experiment resulting in 9 independent samples for rice and 9 independent samples for Arabidopsis. The RNA was isolated using the Qiagen RNeasy Plant RNA isolation kit and DNase treated using both the Qiagen on-column DNase digestion as well as the Ambion Turbo DNase treatment exactly as carried out in Narsai et al., 2007 [55].

### QRT-PCR analysis

Details of the primer sequences, amplicon lengths and RNA to qRT-PCR quality checks for each of the genes analysed are shown in Additional file 1, Table S8. Overall, the procedures for RNA isolation and qRT-PCR for both rice and Arabidopsis were carried out as described in a recent rice reference gene study [81]. Specifically, transcript abundance for each gene was measured using the SYBR green I master (Roche, Sydney) with the Roche LC480 machine. Each control and treated sample was analysed in biological triplicate, using individual plants in order to test for reproducibility. Isolated RNA was quantitated using a Nanodrop spectrophotometer and 1 μg of total RNA was reverse transcribed using the Bio-Rad^® ^(Sydney) iScript reverse transcription kit, according manufacturer's instructions. In parallel to each sample, another 1 μg of RNA was used for the same reverse transcription reaction, with the exception of the addition of the reverse transcriptase enzyme (no RT samples) in order to confirm no DNA contamination. The cDNA and no RT samples were then purified using the Qiagen^® ^PCR purification kit, according to manufacturer's instructions. For the qRT-PCR analysis, 1 μl of this purified cDNA (diluted 1 in 10) for each sample was analysed as well as 1 μl of the undiluted no RT samples. Analysis of the no RT samples in this way allows the detection of any genomic DNA contamination. In order to analyse the data produced by the qRT-PCR quantitation, the comparative Ct method was employed. This method relies on comparison of the threshold cycle numbers of the treated vs. control samples and has been used previously to analyse qRT-PCR data [81]. An outline/checklist for qRT-PCR data presentation has been generated based on a template provided in [82] and this shown in Additional file 1, Table S8C.

## Abbreviations

ABA: abscisic acid; AGI: Arabidopsis gene identifier; APX: ascorbate peroxidase; CAREs: *cis*-acting regulatory element(s); DEG: differentially expressed gene; DHAR: dehydroascorbate reductase; FDR: false discovery rate; GA: gibberellins; GC-RMA GC: content based Robust Multi-array Average; GR: glutathione reductase; MDHAR: monodehydroascrobate reductase; NS: Hbs non-symbiotic haemoglobin; PPDE: Posterior Probability of Differential Expression; qRT-PCR: Quantitative Real Time Polymerase Chain Reaction; TAIR: The Arabidopsis Information Resource; TF: transcription factor; TIGR: The Institute of Genomic Research; UTR: untranslated region

## Authors' contributions

RN carried out all the data analysis. IC helped with the sequence analysis. JW oversaw the analysis, design and implementation. RN and JW drafted the manuscript. All authors read and approved final manuscript.

## Supplementary Material

Additional file 1 **Additional Figures showing overview of expression, common and distinct stress responsive genes, and putative motif analysis.** Figure S1. Overview of expression. A) Number of genes never expressed, always expressed and expressed on average across all the rice microarrays analysed in this study. B) The number of transcripts expressed across the increasing number of samples analysed. Figure S2. Defining common and exclusive stress responsive genes. The number of genes significantly (p < 0.05, PPDE > 0.96) increasing (red bordered boxes) and decreasing (blue bordered boxes) in abundance in rice and Arabidopsis under A) drought, B) salt, C) cold and D) heat treated plants are shown. For each stress, the number of transcripts significantly up/down-regulated in abundance is shown on a Venn diagram as follows; the number of transcripts with no orthologues (lightest shade), with known orthologues (darker shade) and the number of transcripts which were orthologous and showing a common response rice and Arabidopsis (darkest shade). Figure S3. Visualisation of transcripts showing differential regulation between Arabidopsis and rice. A) The FUNCAT breakdown comparison of core and oppositely regulated genes compared to all the genes changing under the respective stress. For example, the FUNCAT analysis was carried out to determine over/under-represented FUNCATs in the 811 core down-regulated genes in rice and Arabidopsis (blue) and compared to the total number of genes down-regulated under drought (dark grey). In this way, over/under-represented FUNCATs for the transcripts regulated in the same/opposite way could be analysed. B) The significant fold-changes of transcripts for control vs. treated, were log transformed and displayed on a custom Figure using the MapMan tool, changes in abundance are represented by shading where the colour saturates at a log2 FC value of 2.5 (i.e. a >5-fold change). The transcript abundance changes compared to the controls are shown for drought (blue bordered boxes), salt (yellow bordered boxes), cold (pink bordered boxes) and heat (green bordered boxes) treated rice and Arabidopsis samples. Transcripts which were orthologous and responding in the same way (up/down) between Arabidopsis (A) and rice (R) were defined as "common A & R" and given that the conservation in response, only one set of values were displayed i.e. for rice only. Where genes were oppositely regulated, the transcript abundance changes for rice (Opposite R) were only visualised. Figure S4. Defining core stress responsive gene expression. The number of genes significantly (p < 0.05, PPDE > 0.96) increasing (i - Abiotic core - UP) and decreasing (ii - Abiotic core - DOWN) in abundance in A) rice and B) Arabidopsis under drought, salt, cold and heat treated plants are shown. Figure S5. Phylogenetic analysis of A) the SEN1 encoding genes in response to cold and heat stress B) the AP2-Dreb family in response to cold and C) an HSF sub-family in response to heat, in both rice and Arabidopsis. Gray circles indicate closely related genes that showed opposite transcript responses. Fold changes in response to heat and/or cold are shown as coloured boxes where the colour of the font indicates up-regulation (red) or down-regulation (blue). Figure S6. Analysis of putative motifs for core orthologous genes showing common response under abiotic stress. A) The occurrence of all possible 6-mers was calculated in each of the orthologous up-regulated and down-regulated subsets for rice and Arabidopsis. The occurrence of each motif in each subset was made relative to the occurrence of that motif in the respective genome. Thus, the heat map intensity represents over (red) or under (blue) representation of putative motifs compared to the genome. From the 4,096 possible 6-mers, only the putative motifs present 20% more or less often than the percentage presence in the genome were visualised e.g. a value of 1.2 indicates that this motif in the respective subset occurs 20% more than the occurrence of this motif in the genome, whilst a value of 0.8 indicates that this motif occurs 20% less than the percentage occurrence in the genome. Examples of conserved over-represented motifs within different stress subsets are indicated in the black boxes. B) The top 3 over-represented motifs from each of the up/down regulated subsets are indicated on the heatmap by the coloured boxes. Note that motifs over-represented in the UP and DOWN regulated sets are indicated by an asterisk *. Click here for file

Additional file 2 **Additional Tables detailing the arrays analysed, FUNCAT statistics and full differential expression analyses. **Table S1. Details of the development/tissue/stress/hormone experiments carried out, the publications addressing these microarrays, the GEO or MIAME Geneexpress accessions, number of biological replications, experimental descriptions (as in Table 1.), the sample details, cultivar, tissue, age and treatment time (where relevant and available) are shown below. Table S2. FUNctional CATalogue (FUNCAT) information from Figure 1. The frequency of transcripts in each FUNCAT and the respective percentages are shown. For each FUNCAT, a z-score statistic and associated p-value was calculated in comparison to the genome. Table S3. Details of the development/tissue/stress/hormone experiments carried out, the publications addressing these microarrays, the GEO or MIAME Geneexpress accessions, number of biological replications, (as in Table 1.), the sample details, genotype, tissue, age and treatment time (where relevant and available), photoperiod and growth substrate are shown below. Table S4. The Arabidopsis microarray probeset identifier (Array element), Annotation (TAIR9) and Locus identifiers (Arabidopsis Gene Identifier - AGI) are annotated. For each stress data subset, the fold change, p-value associated with that fold change and PPDE (< p) (false discovery rate correction) are shown. Table S5. The rice microarray probeset identifier (Probeset ID), TIGR6 gene identifier and TIGR6 putative function are annotated. For each stress data subset, the fold change, p-value associated with that fold change and PPDE (< p) (false discovery rate correction) are shown. Table S6. Lists of genes that have rice/Arabidopsis orthologues and are responsive in a similar manner i.e. i) down in rice and Arabidopsis or ii) up in rice and Arabidopsis or opposite manner i.e. iii) down-regulated in rice, whilst the Arabidopsis orthologue for that gene/s is up-regulated or iv) up-regulated in rice, whilst the Arabidopsis orthologue/s for that genes is down-regulated). TIGR gene identifier and AGIs are shown for rice and Arabidopsis, respectively. Table S7. Analysis of transcription factor families. Z-score analysis of all the families shown in Figure 6. The frequency of transcripts from each family in each subset and the respective percentage is shown. For each family in each subset, a z-score statistic and associated p-value was calculated (p-values shown) in comparison to the genome. Significant over/under-representation (p < 0.01) is indicated in red (over-represented) or blue (under-represented). Percentages underlined in red/blue represent over/under-representation at p < 0.02. Table S8. Genes analysed by qRT-PCR. Primer sequences and amplicon lengths (bp- base pairs) are shown for the respective rice (ST 8A) and Arabidopsis (ST 8B) gene identifiers (TIGR identifiers for rice and AGIs for Arabidopsis) for the genes analysed by qRT-PCR (presented in Figure 5B). The _F at the end of each identifier denotes the forward primer, whilst the R_ denotes the reverse primer. ST 8C shows a checklist outlining the RNA to qRT-PCR quality/methodology as described in [82]. Click here for file
