# Supplementary material for: Common and distinct organ and stress responsive transcriptomic patterns in Oryza sativa and Arabidopsis thaliana
Source: BMC Plant Biol. 2010 Nov 24;10:262. doi: 10.1186/1471-2229-10-262 (PMC3095337; doi:10.1186/1471-2229-10-262)
Supplement: Additional file 1 — Additional Figures showing overview of expression, common and distinct stress responsive genes, and putative motif analysis. Figure S1. Overview of expression. A) Number of genes never expressed, always expressed and expressed on average across all the rice microarrays analysed in this study. B) The number of transcripts expressed across the increasing number of samples analysed. Figure S2. Defining common and exclusive stress responsive genes. The number of genes significantly (p < 0.05, PPDE > 0.96) increasing (red bordered boxes) and decreasing (blue bordered boxes) in abundance in rice and Arabidopsis under A) drought, B) salt, C) cold and D) heat treated plants are shown. For each stress, the number of transcripts significantly up/down-regulated in abundance is shown on a Venn diagram as follows; the number of transcripts with no orthologues (lightest shade), with known orthologues (darker shade) and the number of transcripts which were orthologous and showing a common response rice and Arabidopsis (darkest shade). Figure S3. Visualisation of transcripts showing differential regulation between Arabidopsis and rice. A) The FUNCAT breakdown comparison of core and oppositely regulated genes compared to all the genes changing under the respective stress. For example, the FUNCAT analysis was carried out to determine over/under-represented FUNCATs in the 811 core down-regulated genes in rice and Arabidopsis (blue) and compared to the total number of genes down-regulated under drought (dark grey). In this way, over/under-represented FUNCATs for the transcripts regulated in the same/opposite way could be analysed. B) The significant fold-changes of transcripts for control vs. treated, were log transformed and displayed on a custom Figure using the MapMan tool, changes in abundance are represented by shading where the colour saturates at a log2 FC value of 2.5 (i.e. a >5-fold change). The transcript abundance changes compared to the controls are shown for drought (blue [file 1471-2229-10-262-S1.PDF]

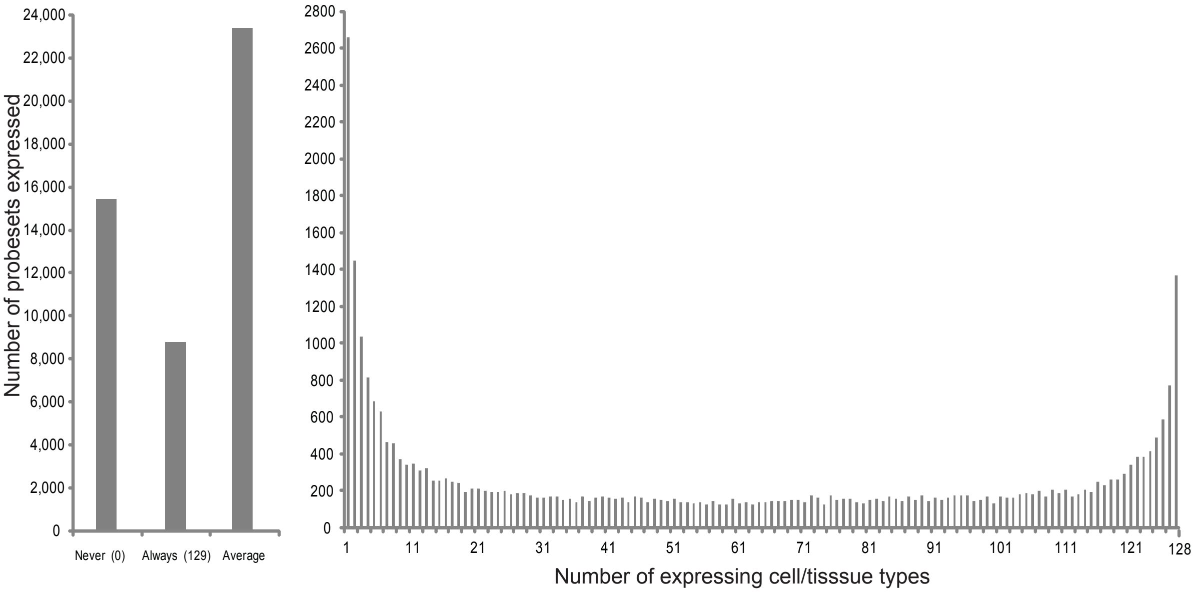

Supplementary Figure 1. Overview of expression. A) Number of genes never expressed, always expressed and expressed on average across all the rice microarrays analysed in this study. B) The number of transcripts expressed across the increasing number of samples analysed.

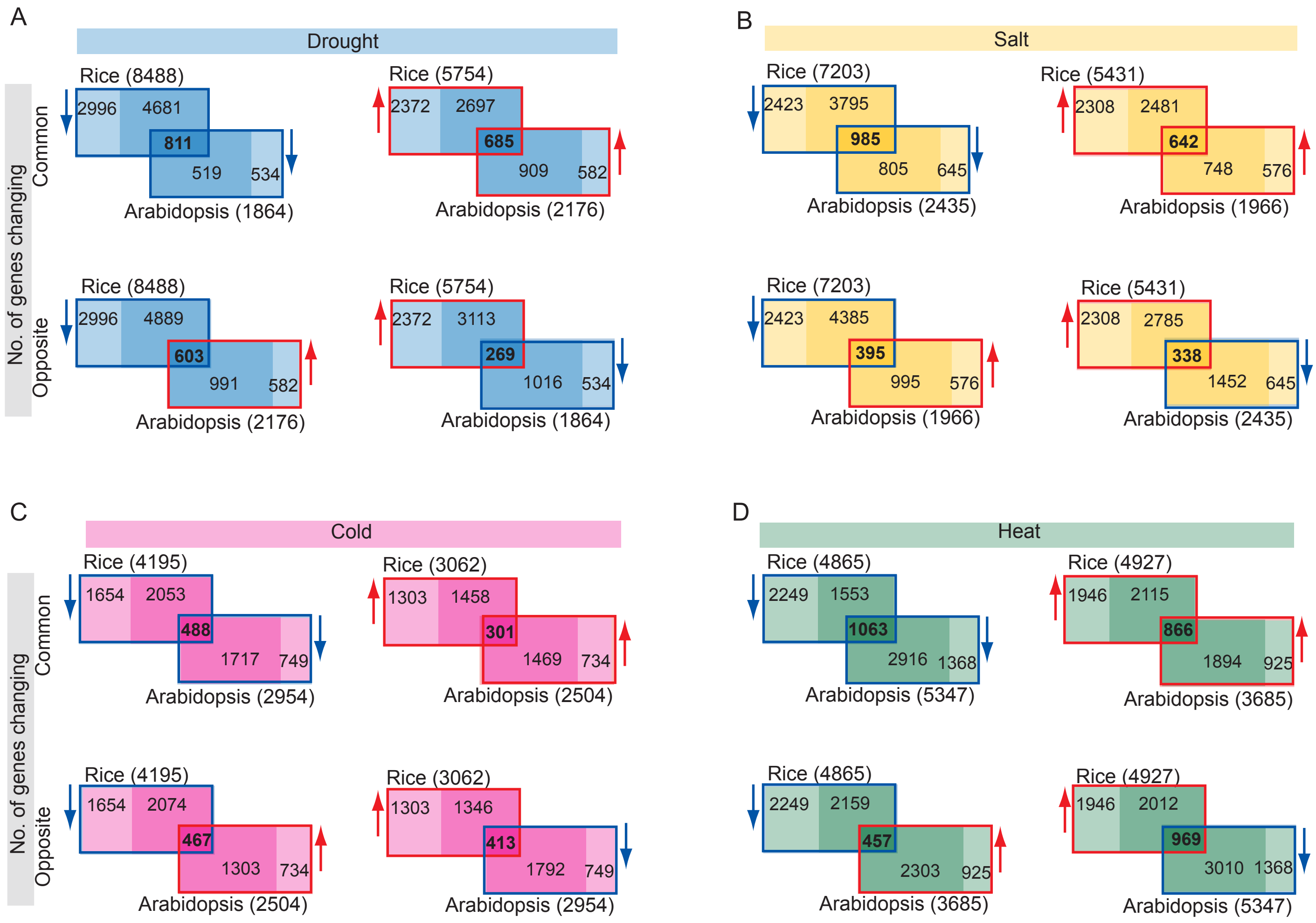

Supplementary Figure 2. Defining common and exclusive stress responsive genes. The number of genes significantly ($p<0.05$, PPDE>0.96) increasing (red bordered boxes) and decreasing (blue bordered boxes) in abundance in rice and Arabidopsis under A) drought, B) salt, C) cold and D) heat treated plants are shown. For each stress, the number of transcripts significantly up/down-regulated in abundance is shown on a Venn diagram as follows; the number of transcripts with no orthologues (lightest shade), with known orthologues (darker shade) and the number of transcripts which were orthologous and showing a common response rice and Arabidopsis (darkest shade).

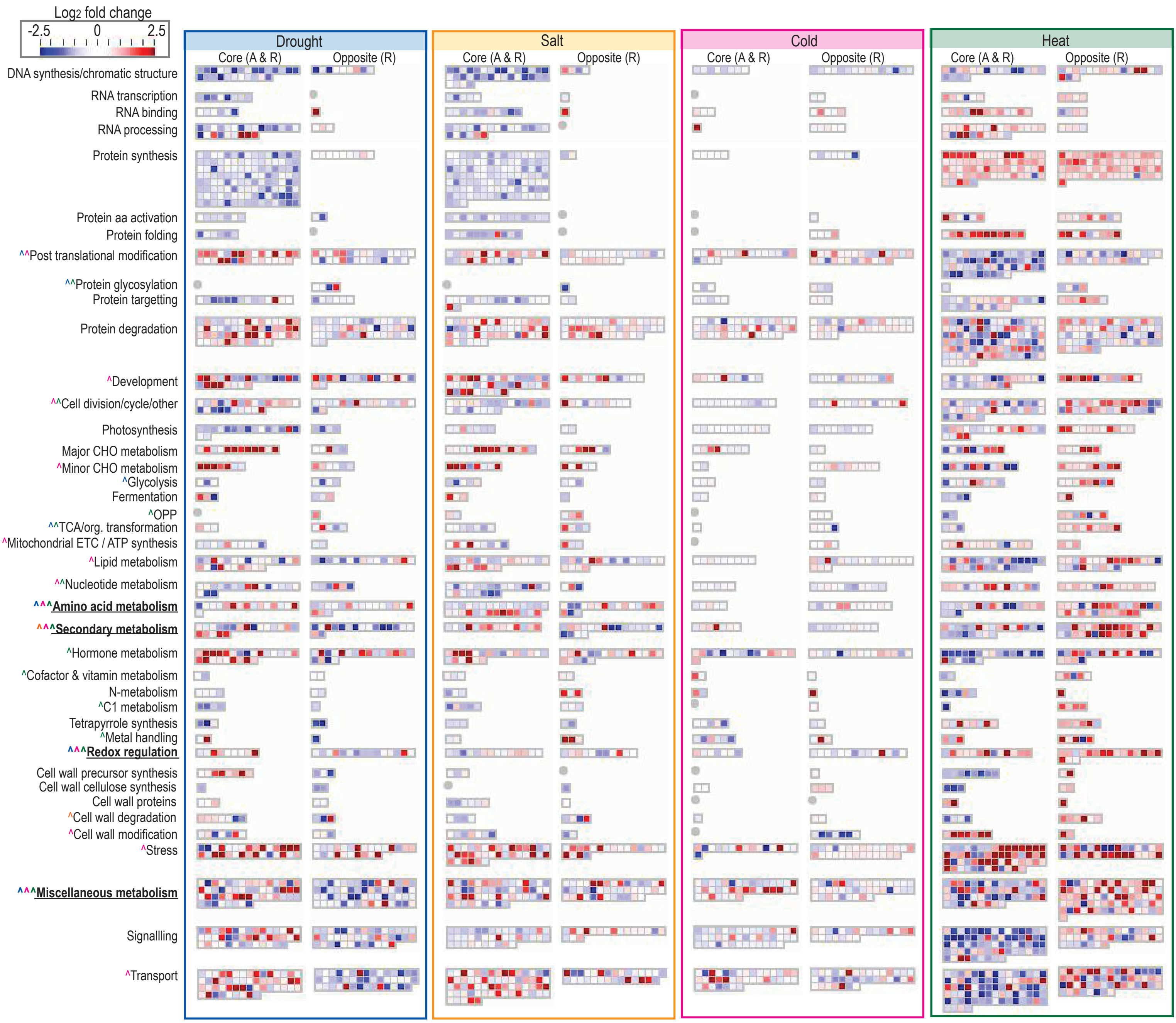

Supplementary Figure 3. Visualisation of transcripts showing differential regulation between Arabidopsis and rice. A) The FUNCAT breakdown comparison of core and oppositely regulated genes compared to all the genes changing under the respective stress. For example, the FUNCAT analysis was carried out to determine over/under-represented FUNCATs in the 811 core down-regulated genes in rice and Arabidopsis (blue) and compared to the total number of genes down-regulated under drought (dark grey). In this way, over/under-represented FUNCATs for the transcripts regulated in the same/opposite way could be analysed. B) The significant fold-changes of transcripts for control vs. treated, were log transformed and displayed on a custom figure using the MapMan tool, changes in abundance are represented by shading where the colour saturates at a log2 FC value of 2.5 (i.e. a >5-fold change). The transcript abundance changes compared to the controls is shown for drought (blue bordered boxes), salt (yellow bordered boxes), cold (pink bordered boxes) and heat (green bordered boxes) treated rice and Arabidopsis samples. Transcripts which were orthologous and responding in the same way (up/down) between Arabidopsis (A) and rice (R) were defined as “common A & R” and given that the conservation in response, only one set of values were displayed i.e. for rice only. Where genes were oppositely regulated, the transcript abundance changes for rice (Opposite R) were only visualised.

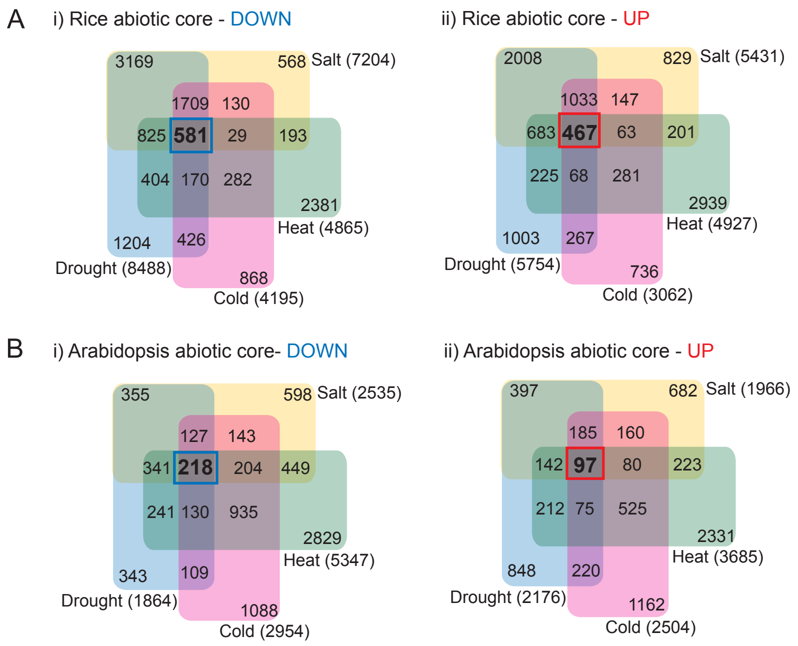

Supplementary Figure 4. Defining core stress responsive gene expression. The number of genes significantly ($p<0.05$, PPDE>0.96) increasing (i - Abiotic core – UP) and decreasing (ii - Abiotic core – DOWN) in abundance in **A)** rice and **B)** Arabidopsis under drought, salt, cold and heat treated plants are shown.

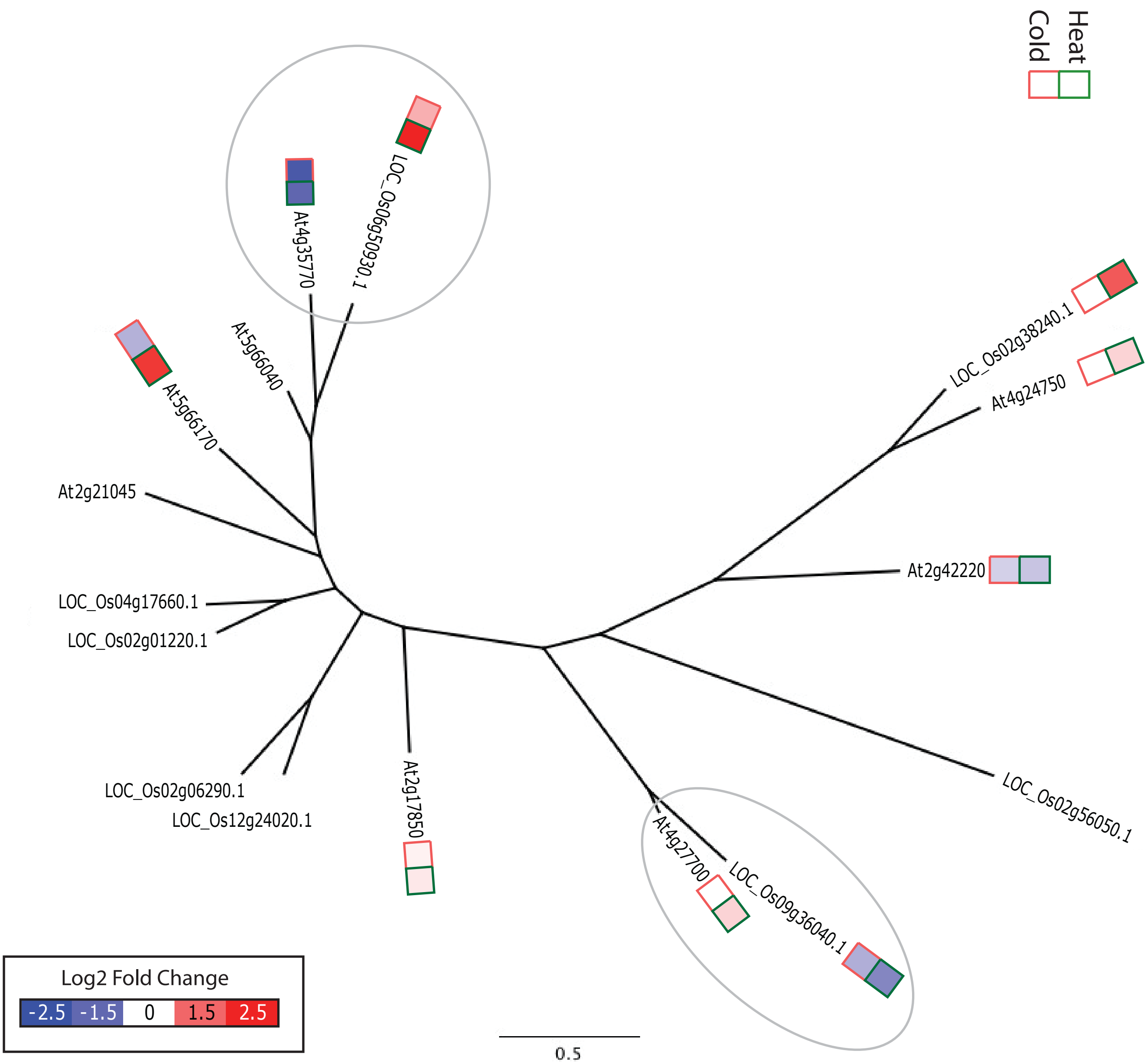

Supplementary Figure 5A. Phylogenetic analysis of the SEN1 gene and close orthologues in response to cold and heat. Gray circles indicate closely related genes that showed opposite transcript responses. Fold changes in response to heat and/or cold are shown as coloured boxes where the colour of the font indicates up-regulation (red) or down-regulation (blue).

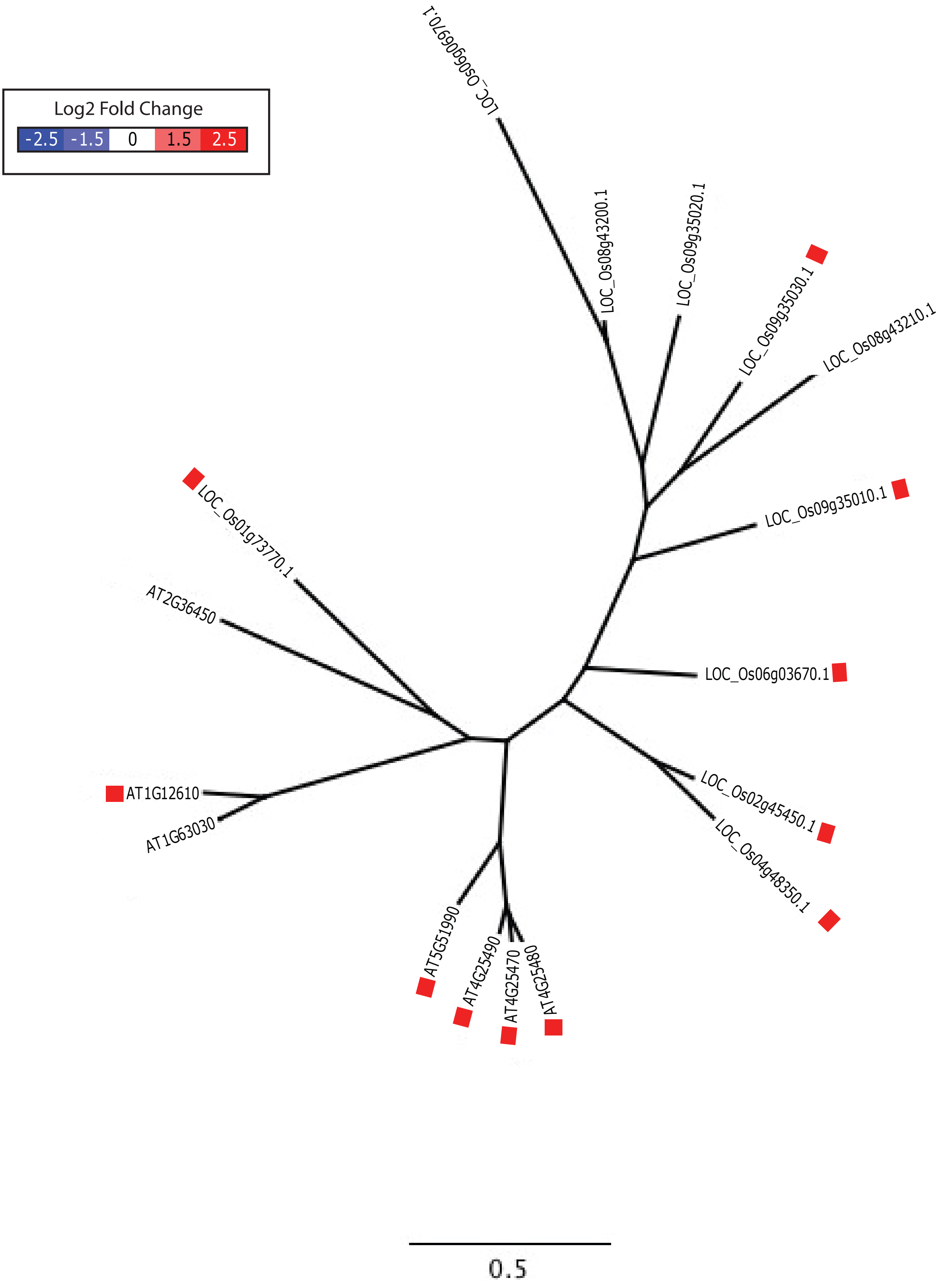

Supplementary Figure 5B. Phylogenetic analysis of an AP2-DREB sub-family in response to cold. Fold changes in response to heat and/or cold are shown as coloured boxes where the colour of the font indicates up-regulation (red) or down-regulation (blue).

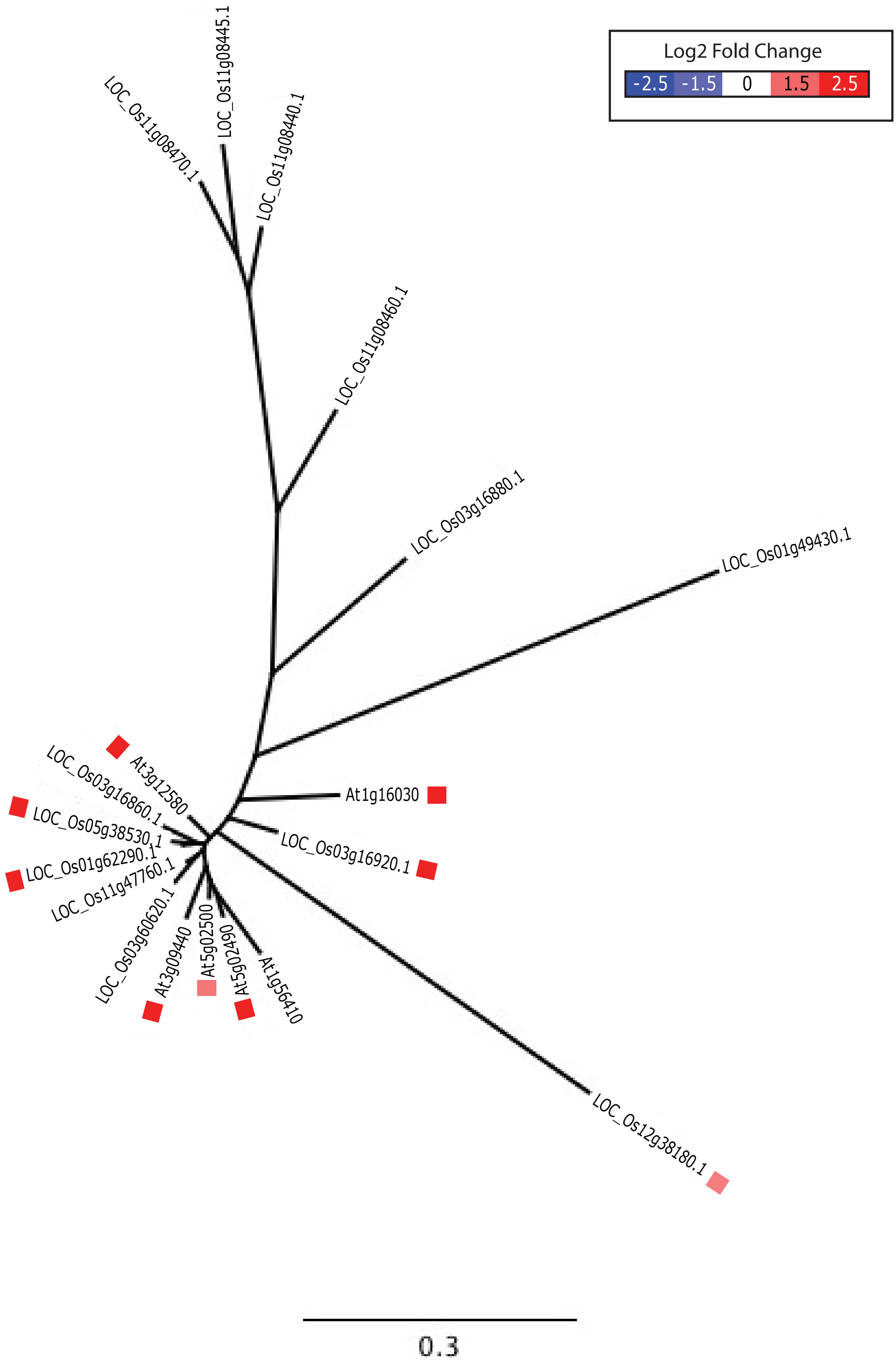

Supplementary Figure 5C. Phylogenetic analysis of an HSF sub-family in response to heat. Fold changes in response to heat and/or cold are shown as coloured boxes where the colour of the font indicates up-regulation (red) or down-regulation (blue).

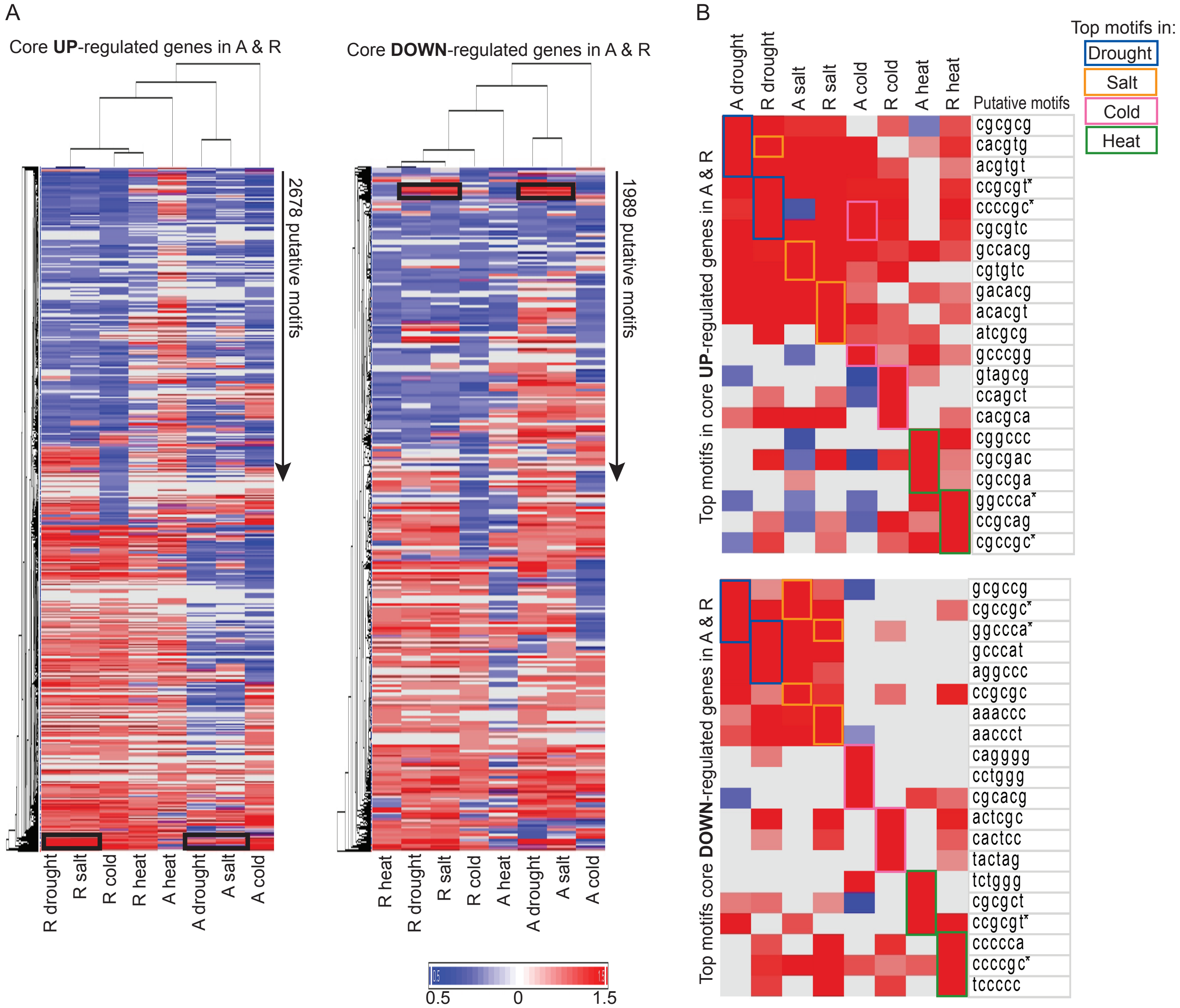

Supplementary Figure 6. Analysis of putative motifs for core orthologous genes showing common response under abiotic stress. A) The occurrence of all possible 6-mers were calculated in each of the orthologous up-regulated and down-regulated subsets for rice and Arabidopsis. The occurrence of each motif in each subset was made relative to the occurrence of that motif in the respective genome. Thus, the heat map intensity represents over (red) or under (blue) representation of putative motifs compared to the genome. From the 4,096 possible 6-mers, only the putative motifs present 20% more or less often than the percentage presence in the genome were visualised e.g. a value of 1.2 indicates that this motif in the respective subset occurs 20% more than the occurrence of this motif in the genome, whilst a value of 0.8 indicates that this motif occurs 20% less than the percentage occurrence in the genome. Examples of conserved over-represented motifs within different stress subsets are indicated in the black boxes. B) The top 3 over-represented motifs from each of the up/down regulated subsets are indicated on the heat-map by the coloured boxes. Note that motifs over-represented in the UP and DOWN regulated sets are indicated by an asterisk *.
